# Determinants of selective domains of cognitive impairment among diabetes mellitus patients: a primary health care setting-based study in India

**DOI:** 10.1038/s41598-025-28613-2

**Published:** 2025-12-29

**Authors:** Nipun Vattathode Murali, Nitin Joseph, Vasudha Kadikadka Gangadhara

**Affiliations:** 1 Department of Community Medicine, Kasturba Medical College Mangalore, Manipal Academy of Higher Education, Manipal, India; 2 Department of Psychiatry, Kasturba Medical College Mangalore, Manipal Academy of Higher Education, Manipal, India

**Keywords:** Type 2 diabetes mellitus, Domain-specific cognitive impairment, Determinants, Diseases, Endocrinology, Health care, Medical research

## Abstract

Cognitive impairment (CI) is a known complication of type II diabetes mellitus (T2DM), potentially hindering effective self-care. While many studies have addressed global cognitive decline, few have explored domain-specific impairments in Indian community settings. This study aimed to assess the extent of CI among T2DM patients and identify the prevalence and determinants of specific deficits in working memory; focused, sustained, divided attention, and problem solving. A primary health care setting-based cross-sectional study was conducted among 441 urban T2DM patients aged 30–65 years in southern India. Participants with ≥ 4 years of diabetes mellitus underwent assessment with the Montreal Cognitive Assessment tool. Those scoring < 26 were assessed using standardized tools for distinct cognitive domains: the Auditory Verbal Learning Test, Color Trail Test, Digit Vigilance Test, Tower of London, and Triads Test. Relevant sociodemographic and clinical variables were collected via a validated interview schedule. The chi-square test, Fisher’s exact test, the Mann‒Whitney U test, and binary logistic regression analysis were used to test associations. Of the total 441 participants, 138 (31.3%) had cognitive impairment. Among them, impairments in divided attention, verbal learning, immediate verbal memory, delayed verbal memory, recognition memory, planning and problem-solving ability, sustained attention and processing speed, sustained attention accuracy and impulsivity control, and focused attention and cognitive flexibility were present among 48 (34.7%), 108 (72.8%), 127 (92.0%), 133 (96.3%), 123 (89.1%), 15 (10.8%), 98 (71.0%), 115(83.3%), and 17 (12.3%) patients, respectively. Significant associations were observed between recent random blood sugar levels ≥ 200 mg/dl and impaired verbal learning [adjusted odds ratio (AOR) 2.35 (0.99–5.54), *p* = 0.052); skilled/unskilled occupation [AOR 9.53 (1.28–71.12), *p* = 0.028)], homemaker/unemployed occupation [AOR 5.48 (0.96–31.16), *p* = 0.055], overweight/obese status [AOR 5.05 (1.1–23.08), *p* = 0.037] and impaired immediate verbal memory; joint family status (*p* = 0.053), absence of family history of DM [AOR 4.07 (1.87–14.015), *p* = 0.026] and impaired planning and problem-solving; middle SES and impaired sustained attention (processing speed) [AOR 3.59 (1.35–9.59), *p* = 0.011]; and being married [AOR 8.4 (1.6–44.5), *p* = 0.012], absence of family history of DM [AOR 4.38 (1.43–13.4), *p* = 0.01] and impaired sustained attention (accuracy); and lower SES and impaired focussed attention [AOR 8.96 (1.01–81.85), *p* = 0.05]. One in three individuals with T2DM had cognitive deficits, particularly in the memory and attention domains. These findings underscore the importance of incorporating domain-specific cognitive screening into routine diabetes care among the high-risk groups identified in this study for early intervention and improved management outcomes.

**Supplementary Information:**

The online version contains supplementary material available at 10.1038/s41598-025-28613-2.

## Background

 Diabetes mellitus (DM) is a chronic metabolic disorder known for its complications involving multiple organs, including the brain^1^. Globally, more than half a billion adults currently live with DM, and this figure is projected to exceed 783 million by 2045, mostly affecting low- and middle-income countries^2^. India alone had approximately 77 million DM patients in 2021, which is expected to reach 134 million by 2045, with more than half remaining undiagnosed^3^.

Patients with cognitive impairment (CI) present with deficits in cognitive domains such as attention, memory, language, planning and problem solving that are beyond what is expected with normal aging but not severe enough to qualify as dementia^4^. These impairments are often associated with neuropsychological disturbances involving hippocampal dysfunction, impaired executive control from prefrontal cortex involvement, and disrupted frontoparietal connectivity affecting attentional regulation, driven primarily by metabolic, vascular, or inflammatory changes common in chronic DM^5^. Each cognitive domain may be selectively affected, contributing uniquely to diminished quality of life and complicating self-management of the disease.

Emerging evidence suggests that cognitive decline in T2DM (Type II diabetes mellitus) patients is often domain specific, with more pronounced impairments observed in planning and problem-solving, attention, and memory, rather than a uniform cognitive decline^6^.

Although the Montreal Cognitive Assessment (MoCA) is commonly used, it may not adequately capture these domain-specific impairments, especially in populations with limited education or in older adults, leading to under- or misclassification^7,8^.

As the global prevalence of both DM and CI continues to rise, their frequent coexistence poses a challenge that is often overlooked in routine care^9^. Early detection of domain-specific deficits is critical for guiding individualized management strategies and improving patient outcomes by improving both metabolic and cognitive health. There are very few studies that have researched on domain-specific CI among T2DM patients.

This study addresses this knowledge gap by examining specific cognitive domains in individuals with T2DM. The objectives of this study were to estimate the prevalence of impairments in working memory; focused, sustained, and divided attention; and problem solving and to identify their associated determinants. These findings could lead to the development of targeted interventions that could support holistic DM care models.

## Materials and methods

This primary health care setting-based cross-sectional study was conducted between December 2022 and September 2024 across two primary health centers (PHCs) in the Lady Hill and Bejai ward areas of Mangalore city, which is affiliated with a private medical college in Mangalore, India.

The study was approved by the Institutional Ethics Committee. Written informed consent was obtained from all participants after ensuring their anonymity and the confidentiality of the information obtained from them. The study included patients aged 30–65 years with a confirmed diagnosis of T2DM for ≥ 4 years and permanent residency in the study area. The exclusion criteria were patients with comorbidities such as stroke and other severe systemic illnesses, speech and hearing disabilities, psychiatric disorders, preexisting cognitive disorders, type I DM, and those who were unwilling to participate. On the basis of a previous study performed in Manipal, India^10^, which reported a CI prevalence of 50.5% among T2DM patients, the sample size was calculated to be 400 with 5% absolute precision and a 95% confidence level. When a 10% nonresponse rate was added, the final sample size using the formula 4pq/d^2^ was calculated as 440. Data were collected using a validated semi-structured interview schedule translated into the Kannada language. The schedule was language validated by the process of translation and back translation with the help of two Kannada language experts.

The participants seeking treatment for DM on an outpatient department (OPD) basis and those fulfilling the selection criteria stated above were enrolled in this study using convenience sampling method. The screening for CI was conducted at the PHCs. In circumstances where the screening and domain-specific evaluative procedures could not be completed during OPD hours, home visits were performed with prior consent from the participants to complete the tests.

All domain-specific cognitive assessments were performed by a clinical psychologist trained in neuropsychological testing. This was in collaboration with a resident doctor in Community Medicine who received training in using the tools. The clinical psychologist followed standardized administration protocols and scoring procedures as outlined in the NIMHANS Neuropsychology Battery manual. The involvement of trained staff helped ensure that the data collection was consistent and accurate.

**Fig. 1:**
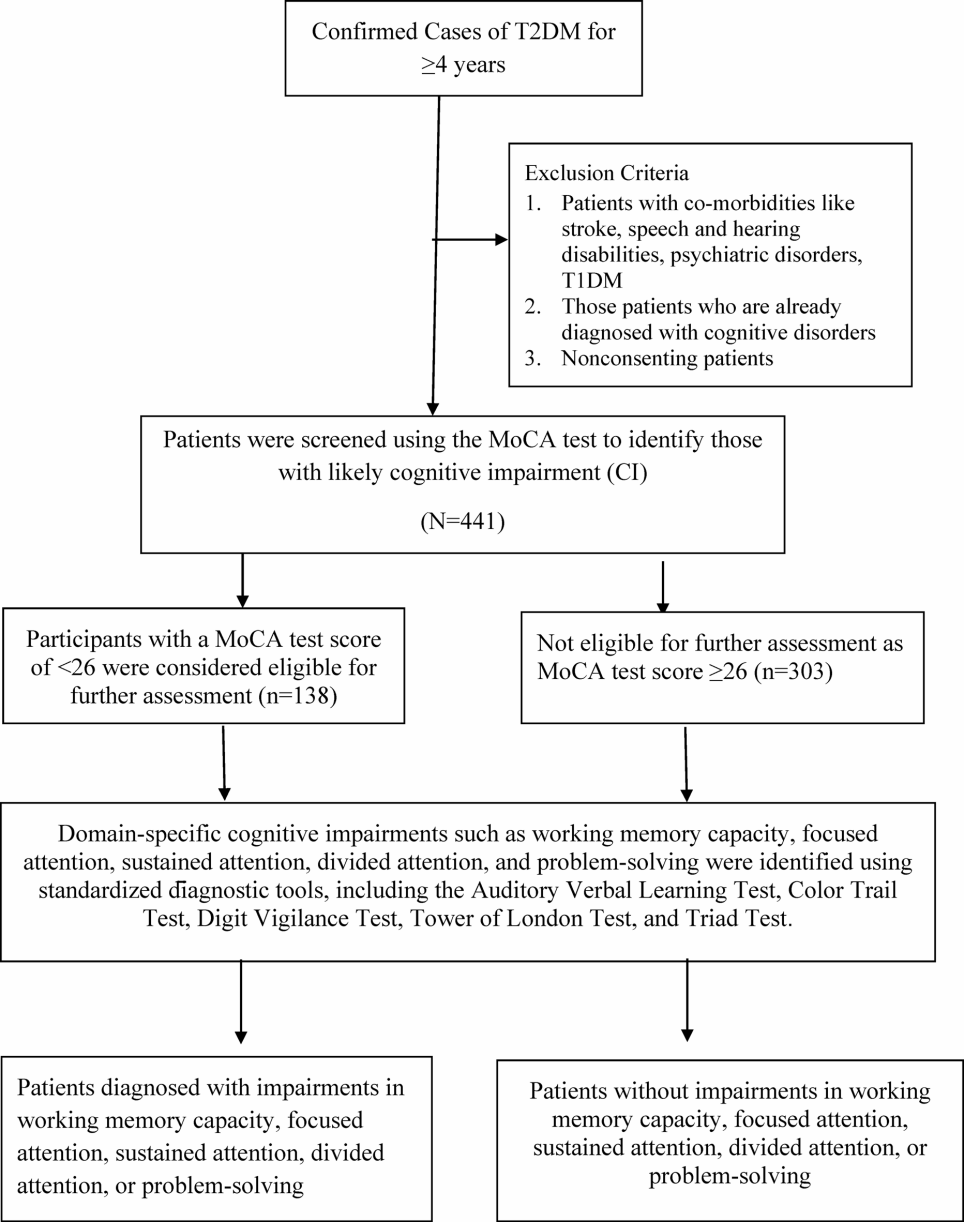
Flowchart of the screening and diagnostic process for identifying domain-specific cognitive impairment (CI) among patients with type 2 diabetes mellitus (T2DM).

Initially, the sociodemographic information and clinical profile of the participants were collected. Next, screening for CI was performed via the Montreal Cognitive Assessment (MoCA) tool^11^. Individuals with MoCA scores below 26 were subsequently assessed using five standardized neuropsychological tests to identify domain-specific CIs. (Fig. 1) Divided attention was measured using the Triads Test, in which participants were required to identify unrelated words while simultaneously recognizing numerals traced on the palm; their performance was evaluated on the basis of the number of errors and time taken to complete the task^12^. The Auditory Verbal Learning Test (AVLT) was employed to assess various components of verbal memory and learning. This included the Trial 5 score, which indicates the participant’s learning capacity after repeated exposure to a word list (List A); immediate recall, which assesses memory retrieval following interference from a secondary list (List B); delayed recall, which is administered after a 20-minute interval to evaluate long-term retention; and the recognition score, which tests the ability to differentiate previously learned words from distractors^13^. Planning and problem-solving were evaluated using the Tower of London task, where participants attempted to solve problems in the fewest possible moves^14^. Sustained attention and processing speed were assessed using the digit vigilance test, with total time and error counts serving as performance metrics^15^. The Color Trail Test 2 (CTT 2) was used to measure focused attention and cognitive flexibility on the basis of the time required to complete the sequencing task^16^.

For each test, raw scores were converted to percentile ranks age- and education-adjusted normative data from the NIMHANS Neuropsychology Battery. Scores below the 15th percentile were classified as indicative of impairment in the respective cognitive domain^12^.

Anthropometric assessments of waist circumference, hip circumference, weight, and height were performed using standardized measurement protocols^17^. Body mass index (BMI) was classified according to the Asia–Pacific classification. According to this classification, underweight, normal, overweight, and obesity were identified among individuals when their BMI was < 18.5, 18.5–22.9, 23–24.9, and ≥ 25 kg/square meter, respectively^18^. The waist‒hip ratio was considered to increase when it was ≥ 0.9 in men and ≥ 0.85 in women according to the WHO classification^17^. A recent glycated haemoglobin (HbA1c) value less than 6.5% was considered an indicator of good sugar control among patients^19^.

The modified Kuppuswamy scale of 2022 was used to assess socioeconomic status (SES) and to categorize the participants as belonging to the “Upper”, “Upper middle”, “Lower middle”, “Upper lower”, or “Lower” SES families^20^.

History of physical activity was considered to be present if the participant indulged in any form of exercises apart from the routine occupation-related physical activities. The details of the comorbidities of the patients were acquired from them and ascertained from the OPD medical records.

Performance on one or more cognitive domains which is below the 15th percentile cutoff based on age- and education-adjusted normative data (on a culturally validated neuropsychological battery) was referred to as CI.^12^

The definition used for each specific cognitive domain has been listed in STable 1.

The data were analyzed via IBM SPSS Statistics Version 29. Descriptive statistics were used to summarize participant characteristics and test scores. Associations were examined using the chi-square test and Fisher’s exact test, with statistical significance set at *p* ≤ 0.05. The Kolmogorov‒Smirnov test was used to test the normality of the distribution of continuous variables. None of the continuous variables, such as age, recent random blood sugar (RBS) values, recent HbA1c values, duration of DM, duration of substance usage, or duration of exercise per week, followed a normal probability distribution. Hence, the Mann‒Whitney U test was used to test association of these variables with impairments in specific cognitive domains among the patients. All those variables associated with domain-specific CIs at a p value of 0.2 or less were further analyzed using binary logistic regression analysis. These variables were age, sex, education, occupation, SES, type of family, marital status, family history of DM, recent RBS values, recent HbA1c value, duration of DM in years, BMI, waist–to-hip ratio, specific comorbidities, and history of physical activity, alcohol usage, smoking or tobacco chewing among the participants, which were adjusted for in the multivariable analysis.

The model-building process involved first identifying all the variables associated with a specific CI by conducting a univariate analysis. Then, all those variables significant with p values ≤ 0.2 for each specific CI were identified and introduced at a time into the domain-specific multivariable analysis. The Hosmer–Lemeshow test was used to test the goodness-of-fit of each model. Nagelkerke’s R squared value was used to indicate the proportion of variance for each model and its predictive accuracy. Then, backward stepwise regression was performed, which eliminated insignificant determinants in a stepwise pattern from the model. The variables in the last step of the model are presented with their adjusted odds ratios (AORs) and their corresponding 95% confidence intervals. Variables with p values ≤ 0.05 were considered significant independent determinants for each domain-specific CI in the multivariable analysis.

## Results

A substantial proportion of the 441 participants were in older age groups, with 155 (35.1%) aged 61–65 years, with a mean age of 56.3 years (SD 7.21) and a median age of 58 years (IQR 52, 62). Males constituted 251 (56.9%) of the study population. In terms of educational attainment, 141 (32.0%) were graduates or postgraduates, and 121 (27.4%) had completed intermediate/diploma-level courses. A total of 111 (25.2%) of the participants were homemakers. The majority of the participants [186 (42.2%)] were of upper middle SES. Most participants were married (93.7%) and lived in nuclear families (81.9%). All participants were urban residents. (Table 1)

**Table 1:** Sociodemographic characteristics of type 2 diabetes mellitus (T2DM) patients (*n* = 441).

Sociodemographic variables	Number	Percentage
Age (years)		
35–40	18	4.1
41–45	19	4.3
46–50	56	12.7
51–55	81	18.4
56–60	112	25.4
61–65	155	35.1
Gender		
Male	251	56.9
Female	190	43.1
Education		
Illiterate	5	1.1
Primary school	16	3.6
Middle school	55	12.5
High school	89	20.2
Intermediate or Diploma	121	27.4
Graduate or Postgraduate	141	32.0
Professional Degree	14	3.2
Occupation		
Unemployed	15	3.4
Unskilled	35	7.9
Semiskilled	90	20.4
Skilled	10	2.2
Clerical	89	20.2
Semiprofessional Professional	66 25	15.0 5.7
Homemaker	111	25.2
Socioeconomic class		
Upper	43	9.7
Upper Middle	186	42.2
Lower Middle	116	26.3
Upper Lower	71	16.1
Lower	25	5.7
Marital Status		
Married	413	93.7
Unmarried	9	2.0
Widow	15	3.4
Divorced/Separated	4	0.9
Type of family		
Nuclear	361	81.9
Joint	67	15.2
Three-generation	13	2.9
Place of Residence		
Urban	441	100.0
Total	441	100.0

The most commonly reported duration of DM was 6–10 years [197 (44.7%)], with a median duration of 6.2 (5, 10) years. Nearly half of the participants (*n* = 213, 48.3%) had recent RBS values between 140 and 199 mg/dL, whereas only 64 (14.5%) had RBS levels below 140 mg/dL. As many as 240 (54.4%) reported monitoring their blood glucose monthly.

On the basis of BMI classification, 202 (45.8%) of the participants were categorized as obese. Central obesity, as indicated by a high waist‒to‒hip ratio, was observed in 397 (90.0%) of the participants. A positive family history of DM was reported by 251(56.9%) of the participants. Comorbid conditions were present in 210 (47.6%) of the patients, with hypertension being the most prevalent [183 (87.1%)] among them.

With respect to treatment, metformin was the most frequently prescribed medication (398, 90.2%), followed by glimepiride (211, 47.8%). One patient was not on any medication for sugar control. (Table 2)

**Table 2:** Clinical characteristics of T2DM patients (*n* = 441).

Clinical characteristics	Number	Percentage
Duration of DM (years)		
4–5 6–10 11–15 > 15	156 197 50 38	35.4 44.7 11.3 8.6
Recent random blood sugar (RBS) values (mg/dl)		
< 140 140–199 > 200	64 213 164	14.5 48.3 37.2
Frequency of Testing RBS		
Biweekly Monthly Once in 1 to 3 months Once In 3 to 6 Months Once in more than 6 months	45 240 108 43 5	10.2 54.4 24.5 9.8 1.1
BMI (kg/m^2^)		
Under weight Normal Overweight Obese	18 134 87 202	4.1 30.4 19.7 45.8
Waist -Hip Ratio		
Normal High	44 397	10.0 90.0
Family history of DM		
Present Absent	251 190	56.9 43.1
Comorbidities in DM Patients		
Present Absent	210 231	47.6 52.4
Comorbidities among participants (*N* = 210)*		
Hypertension Hypercholesterolemia Hypothyroidism Anaemia	183 22 15 2	87.1 10.5 7.1 0.9
Medicines taken by the T2DM patients *		
Metformin Glimepiride Insulin therapy Vildagliptin Glibenclamide Voglibose Sitagliptin Teneligliptin Glipizide No treatment	398 211 28 28 21 14 12 8 7 1	90.2 47.8 6.3 6.3 4.8 3.2 2.7 1.8 1.6 0.2
Total	441	100.0

*Multiple responses.

**Fig. 2:**
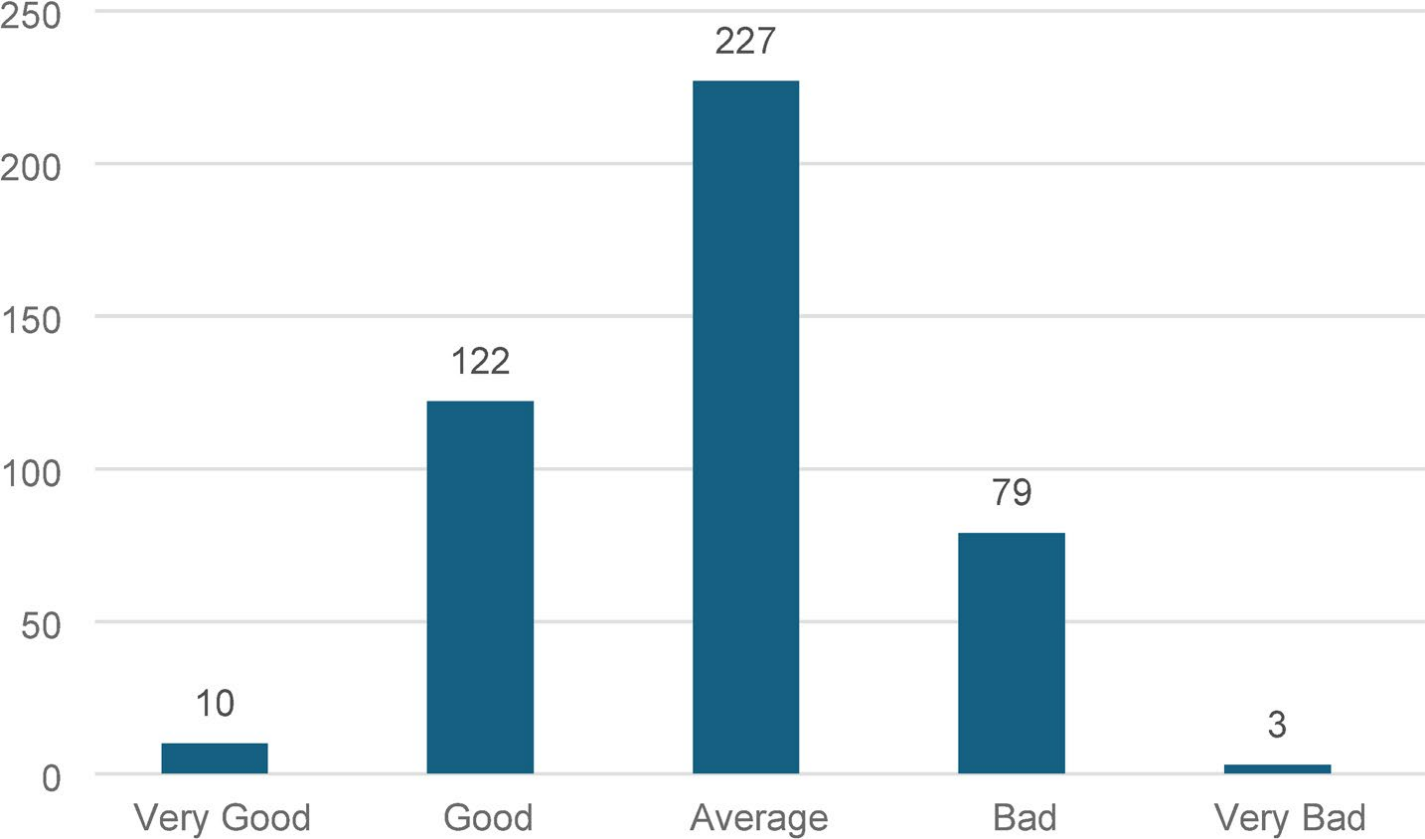
Perception of Type 2 Diabetes Mellitus patient regarding blood sugar control (*N*=441).

Regarding perceived DM control, 227 (51.5%) of the participants rated their control as average. A small proportion rated it as very good (10, 2.3%) or very bad (3, 0.7%). (Fig. 2)

Among the 441 individuals with DM, 165 (37.4%) engaged in any form of physical activity. Among those who exercised, 131 participants provided further details of their exercise practices, such as 92 (70.2%) had sustained this practice for 6–10 years, and 68 (51.9%) reported exercising for 150–300 min per week.

Substance use was reported by 72 (16.3%) of the participants. Among them, alcohol was the most commonly used substance (62, 86.1%), followed by smoking (42, 58.3%) and tobacco chewing (11, 15.3%). (Table 3)

**Table 3:** Lifestyle characteristics of T2DM patients (*n* = 441).

Lifestyle characteristics	Number	Percentage
History of Physical Activity		
Present Absent	165 276	37.4 62.6
Duration of Physical Activity (years) (*n* = 131)		
1–5 6–10 11–15 > 15 years	21 92 8 10	16.0 70.2 6.1 7.7
Frequency of Physical activity (mins/week) (*n* = 131)		
< 150 150–300 > 300	17 68 46	13.0 51.9 35.1
Substance Usage		
Present Absent	72 369	16.3 83.7
Distribution of substance use* (*n* = 72)		
Alcohol Smoking Tobacco chewing	62 42 11	86.1 58.3 15.3
Total	441	100.0

*Multiple responses.

**Fig. 3:**
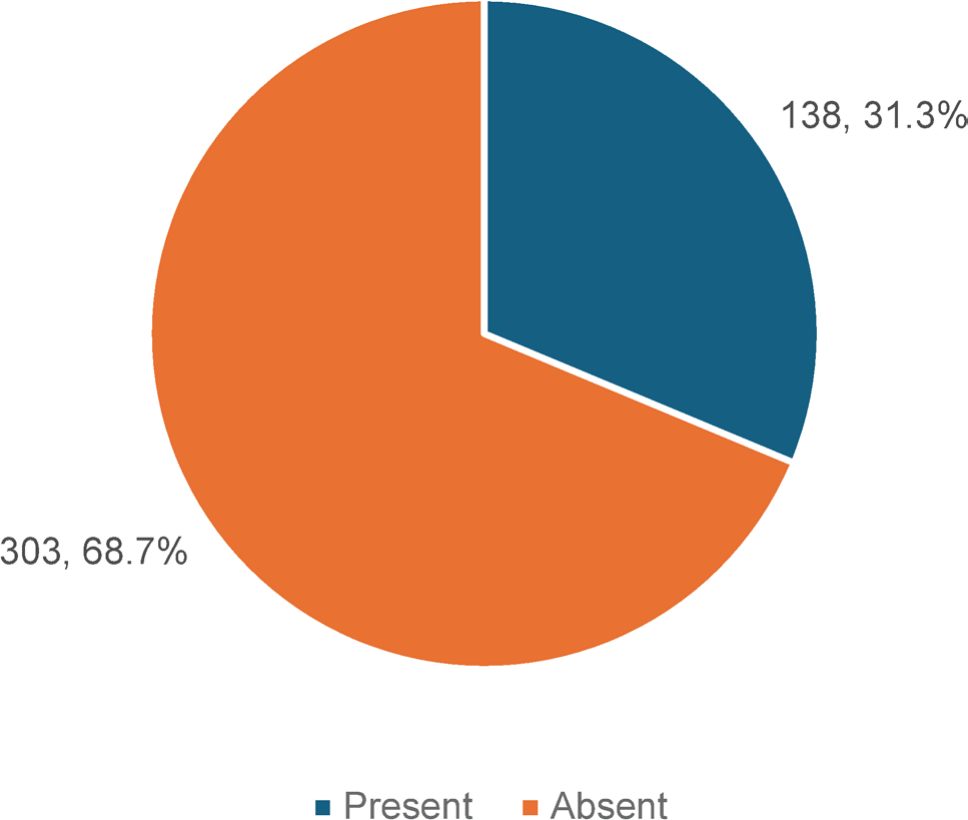
Cognitive Impairment status among the Type 2 Diabetic Mellitus patients (*N*=441).

Among the 441 individuals with DM, 138 (31.3%) had CI based on the MoCA. (Fig. 3)

Among these 138 DM patients with CI, 48 (34.7%) had impaired divided attention based on the TRIAD test. According to both the univariate and adjusted analyses, no sociodemographic or clinical variables were significantly associated with divided attention impairment. Overweight or obese status and smoking were associated with increased odds in both models, but these associations did not reach statistical significance (Tables 4 and 5).

**Table 4:** Association between sociodemographic variables and divided attention among participants with cognitive impairment (*n* = 138).

Characteristics		Divided attention			COR (95% CI)	*P* value	AOR (95% CI)	*P* value
		Impaired	Intact					
		*n* (%)	*n* (%)	Total				
Age (years)	<50	12 (25.1)	16 (17.8)	28	1.00	0.315		
	≥50	36 (74.9)	74 (82.2)	110	0.649 (0.27–1.51)			
Gender	Male	31 (36.9)	53 (63.1)	84	0.786 (0.38–1.62)	0.514		
	Female	17 (31.5)	37 (68.5)	54	1.00			
Education	Up to class 10	27 (31.4)	59 (68.6)	86	0.68 (0.33–1.38)	0.284		
	Beyond Class 10	21 (40.4)	31 (59.6)	52	1.00			
Occupation	Professional	8 (22.9)	27 (77.1)	35	1.00			
	Skilled & Unskilled	23 (38.3)	37 (61.7)	60	2.20(0.813–5.98)	0.228		
	Homemaker/Unemployed	17 (39.5)	26 (60.5)	43	2.098(0.81–5.39)			
Socioeconomic class	Upper	19 (45.2)	23 (54.8)	42	1.00			
	Middle	14 (29.8)	33 (70.2)	47	0.514(0.215–1.23)			
	Lower	15 (30.6)	34 (69.4)	49	0.534(0.226–1.26)	0.233		
Type of Family	Nuclear	37 (34.6)	70 (65.4)	107	1.00			
	Joint	11 (35.5)	20 (64.5)	31	1.04 (0.45–2.40)	0.926		
Marital Status	Married	45 (34.6)	85 (65.4)	130	1.00			
	Unmarried	3 (37.5)	5 (62.5)	8	0.88 (0.20–3.86)	0.868		
Family History of DM	Present	23 (31.5)	50 (68.5)	73	1.36 (0.67–2.74)	0.474		
	Absent	25 (38.5)	40 (61.5)	65	1			
Total		48	90	138				

**Table 5:** Association between clinical characteristics and lifestyle factors and divided attention among participants with cognitive impairment (*n* = 138).

Characteristics		Divided attention		Total	COR (95% CI)	*P* value	AOR (95% CI)	*P* value
		Impaired	Intact					
		*n* (%)	*n* (%)					
Recent RBS value mg/dL	≥ 200	26 (37.7)	43 (62.3)	69	1.29 (0.64–2.61)	0.475		
	< 200	22 (31.9)	47 (68.1)	69	1.00			
HbA1c(*n* = 16)	Good sugar control	0 (0.0)	2 (100.0)	2	1.00	0.997		
	Poor sugar control	1 (7.1)	13 (92.9)	14	0.556 (0.0172–17.9)			
Duration of DM (years)	≤ 10	36 (35.3)	66 (64.7)	102	1.00			
	> 10	12 (33.3)	24 (66.7)	36	0.91 (0.41–2.05)	0.832		
BMI (kg/m2)	Normal/Underweight	12 (25.0)	36 (75.0)	48	1.00		1.00	
	Overweight/Obese	36 (40.0)	54 (60.0)	90	2.00 (0.91–4.35)	0.078	1.99 (0.91–4.37)	0.085
Waist Hip Ratio	Normal	4 (28.6)	10 (71.4)	14	1.00			
	High	44 (35.5)	80 (64.5)	124	1.38 (0.41–4.64)	0.608		
Other Comorbidities among DM Patients								
Hypertension	Hypertensive	22 (33.8)	43 (66.2)	65	0.93 (0.46–1.87)	0.827		
	Normotensive	26 (35.6)	47 (64.4)	73	1.00			
Hypercholesterolemia	Present	1 (14.3)	6 (85.7)	07	3.36 (0.39–28.7)	0.421	–	–
	Absent	47 (35.9)	84 (64.1)	131	1.00			
Hypothyroidism	Present	1 (33.3)	2 (66.7)	3	0.94 (0.08–10.6)	0.995	–	–
	Absent	47 (34.8)	88 (65.2)	135	1.00			
History of Physical Activity	Yes	14 (41.2)	20 (58.8)	34	1.00		–	–
	No	34 (32.7)	70 (67.3)	104	0.90 (0.39–2.09)	0.807		
Alcohol Use	Yes	12 (40.0)	18 (60.0)	30	0.75 (0.33–1.72)	0.498		
	No	36 (33.3)	72 (66.7)	108	1.0			
Smoking Status	Smoker	11 (50.0)	11 (50.0)	22	0.47 (0.19–1.18)	0.102	2.12 (0.83–5.40)	0.114
	Non-Smoker	37 (31.9)	79 (68.1)	116	1.0		1.00	
Tobacco Chewing	Yes	1 (20.0)	4 (80.0)	5	0.46 (0.05–4.21)	0.658	–	–
	No	47 (35.3)	86 (64.7)	133	1.00			
Total		48	90	138				

The Mann‒Whitney U test revealed that age (*p* = 0.0745), recent RBS (*p* = 0.751), duration of substance use (*p* = 0.257), duration of DM (*p* = 0.271), duration of exercise (*p* = 0.416) and recent HbA1c values (*p* = 0.221) were not associated with attention impairment.

Verbal learning, assessed via AVLT trial 5, was impaired in 108 participants (72.8%). According to the univariate analysis, elevated RBS (≥ 200 mg/dL) and overweight/obesity status were associated with increased odds of impairment. However, these associations were not statistically significant in the adjusted model (STable 2, STable 3).

The median duration of DM among patients with verbal learning impairment (*n* = 108) was 6 (5,11) years, whereas it was 7 (5,12) years among those without verbal learning impairment (*n* = 30). These differences in the duration of DM between the two groups were statistically significant according to the Mann‒Whitney U test (*p* = 0.045). However, there was no association of age (*p* = 0.975), recent RBS (*p* = 0.102), duration of substance use (*p* = 0.302), duration of exercise (*p* = 0.553) or recent HbA1c values (*p* = 1.00) with verbal learning impairment.

Immediate verbal memory, measured using the AVLT Immediate Recall scale, was impaired in 127 participants (92.0%). According to the adjusted analysis, overweight or obese individuals had significantly greater odds of impairment (AOR = 5.05; *p* = 0.037), as did those in skilled/unskilled occupations (AOR = 9.53; *p* = 0.028). SES, however, was not associated with immediate verbal memory impairment after adjustment. (STable 4, STable 5).

The median age of patients with immediate verbal memory impairment (*n* = 127) was 59 (52, 63) years, whereas that of those without impairment (*n* = 11) was 63 (58, 64.8) years. These differences in age distribution across the two groups were statistically significant according to the Mann‒Whitney U test (*p* = 0.027). However, there was no association of recent RBS (*p* = 0.301), duration of DM (*p* = 0.707), duration of substance use (*p* = 0.838), duration of exercise (*p* = 0.140) or recent HbA1c values (*p* = 0.505) with immediate memory impairment.

Delayed verbal memory impairment, assessed by the AVLT Delayed Recall subtest, was observed in 133 participants (96.3%). No significant associations were found in either the univariate or adjusted analyses. Although elevated RBS and longer DM duration had greater odds of impairment, these findings were not statistically significant (STable 6, STable 7).

According to the Mann‒Whitney U test, there was no association of age (*p* = 0.945), recent RBS (*p* = 0.257), duration of DM (*p* = 0.536) or recent HbA1c values (*p* = 0.505) with delayed recall.

Impairment in recognition memory was noted in 123 individuals (89.1%) on the basis of AVLT recognition performance. Neither univariate nor adjusted analyses identified any significant predictors. A nonsignificant protective trend was observed among those living in nuclear families (STable 8, STable 9).

The Mann‒Whitney U test revealed that age (*p* = 0.152), recent RBS (*p* = 0.392), duration of substance use (*p* = 0.588), duration of DM (*p* = 0.057), duration of exercise (*p* = 0.056) and recent HbA1c values (*p* = 1.00) were not associated with recognition memory impairment.

Planning and problem-solving ability, measured by the total number of problems solved with minimal moves score, was impaired in 15 participants (10.8%). According to the adjusted analysis, individuals without a family history of DM and those from joint families had significantly greater odds of impairment (STable 10, STable 11).

The median duration of exercise among patients with planning and problem-solving impairment (*n* = 3) was 3 years (IQR here could not be calculated, as there were only 3 values), whereas it was 8 (6,10) years among those without (*n* = 123). These differences in the duration of exercise across the two groups were statistically significant according to the Mann‒Whitney U test (*p* = 0.015).

However, there was no association of age (*p* = 0.891), recent RBS (*p* = 0.965), duration of DM (*p* = 0.662), duration of substance use (*p* = 0.838), duration of exercise (*p* = 0.830) or recent HbA1c values (*p* = 0.505) with planning and problem-solving impairment.

Sustained attention and processing speed, assessed using the DVT total time score, were impaired in 98 participants (71.0%). Middle SES was significantly associated with increased odds of impairment in the adjusted model. No other sociodemographic or clinical variables reached the level of statistical significance (STable 12, STable 13).

The Mann‒Whitney U test revealed that age (*p* = 0.654), recent RBS (*p* = 0.144), duration of DM (*p* = 0.960), duration of substance use (*p* = 0.186), duration of exercise (*p* = 0.965) and recent HbA1c values (*p* = 0.601) were not associated with sustained attention and processing speed impairment.

In relation to sustained attention accuracy and impulsivity control, as measured via the DVT error score, 115 participants (83.3%) experienced impairment. Both univariate and adjusted models revealed that a lack of a family history of DM and being married were significantly associated with greater odds of impairment. Other variables, including age, education, BMI, and comorbidities, showed no significant association (STable 14, STable 15).

The median recent RBS value among patients with sustained attention accuracy and impulsivity impairment (*n* = 115) was 196 (160, 229) mg/dL, whereas it was 212 (186, 267) mg/dL among those without (*n* = 23). These differences in recent RBS across the two groups were statistically significant according to the Mann‒Whitney U test (*p* = 0.055).

However, there was no association of age (*p* = 0.032), duration of DM (*p* = 0.280), duration of substance use (*p* = 1.00), duration of exercise (*p* = 1.00) or recent HbA1c values (*p* = 0.490) with sustained attention accuracy or impulsivity impairment.

Focused attention and cognitive flexibility, assessed through the Color Trail Test 2 (CTT-2), were impaired in 17 participants (12.3%). According to the univariate analysis, participants with lower and middle SES had greater odds of impairment, although these findings were not statistically significant. In the adjusted analysis, lower SES emerged as a significant predictor. Higher odds were also observed in participants with longer durations of DM and among those from the middle SES groups. These trends did not, however, reach the level of statistical significance (STable 16, STable 17).

The median duration of exercise among patients with focused attention and cognitive flexibility impairment (*n* = 17) was 7.11 (6.91, 7.20) years, whereas it was 7 (6.78, 7.22) years among those without focused attention and cognitive flexibility impairment (*n* = 121). These differences in the duration of exercise across the two groups were statistically significant according to the Mann‒Whitney U test (*p* = 0.034).

The median duration of DM was 6 (5, 10) years among patients with focused attention and cognitive flexibility impairment (*n* = 17), whereas it was 10 (6, 17) years among those without the same (*n* = 121). These differences in the duration of DM across the two groups were statistically significant according to the Mann‒Whitney U test (*p* = 0.039). However, there was no association of age (*p* = 0.119), recent RBS (*p* = 0.301), the duration of substance use (*p* = 0.838) or recent HbA1c values (*p* = 0.836) with focused attention and cognitive flexibility among the participants.

## Discussion

CI was identified in 138 out of the 441 individuals with DM in the present study, yielding a prevalence of 31.3%, with memory and attention being the most commonly affected domains. These impairments are influenced by metabolic, social, and familial factors. In another study done at Rural Health and Training Centre in Mandur, Goa, India, 46.5% of DM patients had CI^21^. In two other studies performed among European cohorts comprising an elderly DM population, CI was observed in 49%^22^ and 59.1%^23^ of the participants. High proportion of CI across these diverse settings reinforce the role of DM, in addition to advanced age, as a key risk factor for CI and highlight the relevance of domain-specific screening for early intervention.

In this study, divided attention impairment was identified in 34.7% of individuals with T2DM-related CI. Owing to the ability to manage multiple tasks simultaneously, divided attention is particularly sensitive to metabolic disturbances and age-related decline. Research has shown similar deficits in neurological conditions with subcortical involvement, which may share pathophysiological overlap with DM-related cognitive decline^24^. For example, divided attention is significantly impaired in subcortical ischemic vascular disease, a condition that shares similar pathophysiological mechanisms with diabetic small vessel disease^25^.

In this study, sustained attention impairments were highly prevalent among DM patients, with 83.3% showing reduced accuracy and 71.0% displaying slower processing speed. These findings highlight a marked vulnerability in attentional control among the DM population. Accuracy impairments are consistent with prior evidence linking attentional lapses to cerebral microvascular damage and disrupted white matter integrity affecting frontoparietal networks^26^. Similarly, slower processing speed may reflect delayed reaction times and reduced neural efficiency, which are often attributed to subcortical ischemic changes and chronic hyperglycemia^27^.

Sociodemographic factors played a notable role in CI among the participants in the present study. Participants without a family history of DM were more likely to exhibit sustained attention deficits, possibly due to lower health awareness and delayed engagement in preventive care. In contrast, individuals aware of their familial risk may adopt healthier lifestyles and benefit from earlier diagnosis, potentially mitigating cognitive decline^28^. Marital status also showed a protective association with the unmarried participants having lower odds of impairment compared to their married counterparts. This may reflect lower exposure to chronic caregiving stress and psychosocial strain, which are known to impact cognitive health, particularly in metabolically vulnerable populations^29^.

Focused attention impairment was observed in only 12.3% of the participants, suggesting relative preservation of this domain in the earlier stages of cognitive decline. Focused attention, which involves maintaining task engagement and filtering distractions, may remain intact longer due to later involvement of prefrontal regions^6^. This is supported by evidence showing that cognitive flexibility and set-shifting, which rely on frontal lobe engagement, tend to decline more noticeably in advanced stages of DM-related cognitive dysfunction^30^. In a prospective cohort study conducted in New York, USA, Palta et al.^31^ observed that 54% of older adults with T2DM demonstrated deficits in focused and executive attention. This was much greater than the findings of the present study. This could be because the higher mean age of the participants in the study by Palta et al.^31^ which was 73 years in comparison to 56.3 years among the participants in this study. These findings suggest that attention-related impairments are more prevalent in older populations with longer disease durations.

SES status was also significantly associated with cognitive outcomes. Participants from lower SES backgrounds had higher odds of focused attention impairment, likely reflecting cumulative disadvantage in cognitive reserve, educational attainment, chronic stress exposure, and access to healthcare^32,33^. Additionally, a longer duration of DM was associated with increased impairment, reinforcing the cumulative impact of chronic metabolic dysregulation on attention-related brain systems^34^.

Our findings indicate that verbal episodic memory, especially delayed recall, represents the most impaired cognitive domain in individuals with T2DM, with impairment observed in 96.3% of participants. This prevalence is considerably higher than that reported in a primary care memory clinic study performed in Amsterdam, Netherlands, by Groeneveld et al.^22^, where 30% of participants with T2DM presented with memory impairment. Similarly, Palta et al.^31^ observed memory dysfunction in 33% of older adults with T2DM. In a hospital-based study performed in Iran by Heidari Gorji et al.^35^, the proportion of T2DM patients with memory impairment was 59%. The wide variation in the prevalence of memory dysfunction observed across different studies could be a result of differences in the assessment scale used, differing inclusion criteria of participants with DM in terms of age and duration of DM, and demographic and social differences prevailing in various study settings. However, in the Iranian study, there was no association between the duration of DM and the presence of memory impairment among T2DM patients, which was in accordance with the findings of this study^35^.

Immediate recall impairment was significantly more likely in overweight or obese individuals, which is consistent with evidence that adiposity-related inflammation and insulin resistance impair memory circuits^36,37^. The occupational role also showed an association in this study. Individuals in skilled or unskilled roles were more likely to have memory deficits than professionals. This aligns with the cognitive reserve hypothesis, which suggests that cognitively demanding professions help build resilient neural networks^38^. Interestingly, those from middle SES had lower odds of impairment than those from higher SES, possibly due to lifestyle factors such as reduced physical activity, increased stress loads, and a lack of mental engagement in more affluent populations^39^.

While delayed recall and recognition memory impairments are common, no specific clinical or demographic predictors have emerged. These findings support earlier findings linking these deficits to more global structural changes, particularly in the hippocampus and its connectivity with frontoparietal regions^40^.

Functionally, verbal memory impairments can compromise DM self-management. Difficulties in recalling instructions, adjusting insulin doses, or remembering dietary recommendations may lead to missed medications, poor glucose control, and reduced adherence^41^. These deficits also contribute to lower health literacy, further complicating disease management and increasing the risk of adverse outcomes^42^.

In this study, planning and problem-solving abilities were impaired in 10.8% of individuals with CI, the lowest among all the cognitive domains assessed. This relative preservation may reflect the delayed onset of decline in higher-order cognitive abilities in individuals with T2DM, as skills such as strategic thinking and inhibitory control tend to remain intact during earlier disease stages^43^. Unexpectedly, participants without a family history of DM had greater odds of impairment in this domain. One possible reason is that individuals aware of familial risk may adopt healthier lifestyles or seek earlier screening, potentially delaying cognitive decline^44^. Moreover, participants from joint families were more likely to show impairment in this domain than those from nuclear households were. The added complexity of caregiving responsibilities and shared decision-making in extended families may contribute to chronic stress and cognitive strain^45^.

## Conclusion

This study underscores the significant yet often overlooked burden of domain-specific CI in individuals with T2DM. One in three individuals with T2DM had CI, particularly in the memory and attention domains. Deficits in verbal memory, sustained attention and processing speed are not only common but also closely associated with modifiable factors such as glycemic control, BMI, occupational complexity, and socioeconomic background. These impairments, which are frequently missed by general screening tools, can affect patients’ ability to manage their condition, thereby impacting treatment adherence, health information recall, and decision-making in daily life.

By employing domain-specific neuropsychological assessments, this study identifies critical cognitive challenges with greater precision and emphasizes the opportunity for timely intervention, thereby delaying further cognitive decline.

As T2DM continues to pose a growing global challenge, including in developing countries such as India, early identification of domain-specific cognitive dysfunctions is needed. This needs to be followed by integrating cognitive retraining into routine care to improve both clinical outcomes and cognitive well-being among patients with DM.

### Limitations

This was a cross-sectional study and hence has limitations in establishing a temporal sequence in the association between determinants and impairment in specific cognitive domains. Certain cognitive domains, such as visuospatial skills, naming, language, orientation, and abstraction, were not investigated among the participants because of time constraints. This may have limited the comprehensiveness of the cognitive assessment. Although the 15th percentile cutoff based on age- and education-adjusted normative data from the NIMHANS Neuropsychology Battery fits with prior research, it may not completely match the clinical standards for diagnosing cognitive impairment. This method is used mainly for research purposes and could overestimate or underestimate impairment, especially for individuals close to the cutoff point. Clinical diagnosis usually requires a full assessment, including functional evaluations, clinical interviews, and corroborative history, which are beyond the scope of this study. Therefore, the findings of the present study should be seen as showing domain-specific cognitive vulnerabilities rather than definite clinical diagnoses.

The current study focused on direct links between sociodemographic and clinical factors and cognitive impairments. Moderating and mediating analyses that provide deeper insights, such as how factors such as age or obesity affect these relationships, were not performed in the present study. The investigators plan to include these analyses in future research in this area.

Certain factors, such as family history of dementia, nutritional factors, other confounders of demographics, physical activity, and other cardiovascular diseases, were not collected by the investigators and therefore were not included in the adjusted analysis models.

There was also missing information with respect to certain variables, such as HBA1c values and details of physical activity were not reported by few participants.

The relatively wide confidence intervals reported for certain outcomes suggest uncertainty around their point estimates, reflecting limited precision in the finding despite significant p value.

Additionally, the findings of this study may not be generalizable to other settings or populations beyond the study context.

## Supplementary Information

Below is the link to the electronic supplementary material.

Supplementary Material 1

Supplementary Material 2

## Data Availability

Available in the Figshare repository [https://doi.org/10.6084/m9.figshare.29432528.v1](https:/doi.org/10.6084/m9.figshare.29432528.v1).
