# Supplementary material for: Determinants of selective domains of cognitive impairment among diabetes mellitus patients: a primary health care setting-based study in India
Source: Sci Rep. 2025 Dec 29;15:44891. doi: 10.1038/s41598-025-28613-2 (PMC12748883; doi:10.1038/s41598-025-28613-2)
Supplement: Supplementary file 1 — Supplementary Material 1 [file 41598_2025_28613_MOESM1_ESM.docx]

**STable 1: Definitions for Cognitive Domains**

| **Cognitive Domain** | **Definition and Assessment Tool** |
| --- | --- |
| Divided Attention | The capacity to allocate cognitive resources across several tasks or information sources simultaneously.^1^ |
| Verbal Learning and Memory | Involves the processes of acquiring, storing, and retrieving verbal information over time. It is evaluated based on repeated word-list learning, recognition, immediate recall, and delayed recall trials, capturing attention, encoding, consolidation, and retrieval.^2^ |
| Immediate Verbal Memory | Refers to the ability to recall information within seconds to a few minutes of presentation.^2^ |
| Delayed Verbal Memory | Reflects long-term storage and consolidation of information, typically assessed after 20–30 minutes.^2^ |
| Recognition Memory | Evaluated by asking participants to differentiate target words from distractors following a delay and it measures both memory retention and the ability to distinguish between previously learned and new verbal content.^2^ |
| Planning and Problem Solving | Executive functions involving setting objectives, generating strategies, carrying them out, and monitoring results. Problem solving includes representing the problem, planning, executing the plan, and evaluating the outcome.^3^ |
| Sustained Attention and Processing Speed | Sustained attention is the capacity to maintain focus and respond accurately to relevant stimuli over prolonged periods, reflecting alertness and attentional regulation.^4^  Processing speed refers to the rate at which individuals can perceive, comprehend, and respond to information. It is critical for efficient cognitive performance and for tasks requiring rapid decision-making. ^5^ |
| Focused Attention and Cognitive Flexibility | Focused attention is the ability to respond selectively to specific tactile, visual, or auditory stimuli while filtering out distractions. It represents the most basic level of attentional control and provides the foundation for higher-order functions such as executive functioning and working memory.^6^  Cognitive flexibility is the ability to switch between cognitive sets/strategies following changing contextual demands. As a core executive function, it supports problem solving, decision-making, working memory, inhibition, attention, and monitoring, enabling individuals to adapt their thoughts and actions in novel or unexpected situations.^7^ |

**References**

1. Proctor RW, Vu KPL. Attention: Selection and control in human information processing. [Internet]. Washington: American Psychological Association; 2023 [cited 2025 Sept 8]. Available from: https://content.apa.org/books/17299-000

2. Almkvist O, Rennie A, Westman E, Wallert J, Ekman U. Methods for assessment of Rey Auditory Verbal Learning Test performance in memory clinic patients and healthy adults - at the cross-roads of learning theory and clinical utility. Clin Neuropsychol. 2025 Feb 17;39(2):424–38.

3. Mayer RE. Problem Solving [Internet]. Oxford University Press; 2013 [cited 2025 Sept 8]. Available from: https://academic.oup.com/edited-volume/34404/chapter/291770077

4. Huang CN, Chen HM, Su BY. Type 2 diabetes mellitus: A cross-sectional analysis of glycemic controls and brain health outcomes. Appl Neuropsychol Adult. 2025 Jan 20;1–8.

5. Gettman J. Best Practices in School Neuropsychology: Guidelines for Effective Practice, Assessment, and Evidence‐Based Intervention [Internet]. 1st edn. Miller D, Maricle D, Bedford C, editors. Wiley; 2022 [cited 2025 Sept 8]. Available from: https://onlinelibrary.wiley.com/doi/book/10.1002/9781119790563

6. MacKay-Brandt A. Focused Attention. In: Kreutzer JS, DeLuca J, Caplan B, editors. Encyclopedia of Clinical Neuropsychology [Internet]. New York, NY: Springer New York; 2011 [cited 2025 Sept 8]. p. 1066–7. Available from: http://link.springer.com/10.1007/978-0-387-79948-3_1303

7. Hohl K, Dolcos S. Measuring cognitive flexibility: A brief review of neuropsychological, self-report, and neuroscientific approaches. Front Hum Neurosci. 2024 Feb 19;18:1331960.
