# Supplementary material for: Determinants of selective domains of cognitive impairment among diabetes mellitus patients: a primary health care setting-based study in India
Source: Sci Rep. 2025 Dec 29;15:44891. doi: 10.1038/s41598-025-28613-2 (PMC12748883; doi:10.1038/s41598-025-28613-2)
Supplement: Supplementary file 2 — Supplementary Material 2 [file 41598_2025_28613_MOESM2_ESM.docx]

**STable 2: Association between sociodemographic variables and verbal learning among participants with cognitive impairment (n=138)**

| **Characteristics** | | **Verbal learning** | |  | **COR (95% CI)** | **P value** | **AOR (95% CI)** | **P value** |
| --- | --- | --- | --- | --- | --- | --- | --- | --- |
|  |  | **Impaired** | **Intact** |  |  |  |  |  |
|  |  | **n (%)** | **n (%)** | **Total** |  |  |  |  |
| **Age (years)** | <50 | 23  (82.1) | 5  (17.9) | 28 | 1.00 | 0.577 |  |  |
|  | ≥50 | 85  (77.3) | 25  (22.7) | 110 | 0.739 (0.255–2.14) |  |  |  |
| **Gender** | Male | 66  (78.6) | 18  (21.4) | 84 | 0.955 (0.418–2.18) | 0.912 | - | - |
|  | Female | 42  (77.8) | 12  (22.2) | 54 | 1.00 |  |  |  |
| **Education** | Up to class 10 | 65  (75.6) | 21  (24.4) | 86 | 0.65 (0.27–1.55) | 0.328 | - | - |
|  | Beyond Class 10 | 43  (82.7) | 9  (17.3) | 52 | 1.00 |  |  |  |
| **Occupation** | Professional | 26  (74.3) | 9  (25.7) | 35 | 1.00 | 0.799 | - | - |
|  | Skilled & Unskilled | 48  (80.0) | 12  (20.0) | 60 | 1.38 (0.516–3.72) |  |  |  |
|  | Homemaker/Unemployed | 34  (79.1) | 9  (20.9) | 43 | 1.31 (0.455–3.76) |  |  |  |
| **Socioeconomic class** | Upper | 30  (71.4) | 12  (28.6) | 42 | 1.00 | 0.403 | - | - |
|  | Middle | 39  (83.0) | 8  (17.0) | 47 | 1.95 (0.708–5.37) |  |  |  |
|  | Lower | 39  (79.6) | 10  (20.4) | 49 | 1.56 (0.594–4.09) |  |  |  |
| **Type of Family** | Nuclear | 85  (79.4) | 22  (20.6) | 107 | 0.744 (0.293–1.89) | 0.533 | - | - |
|  | Joint | 23  (74.2) | 8  (25.8) | 31 | 1.00 |  |  |  |
| **Marital Status** | Married | 102 (78.5) | 28 (21.5) | 130 | 1.00 |  | – | – |
|  | Unmarried | 6 (75.0) | 2 (25.0) | 08 | 0.82 (0.16–4.31) | 0.818 |  |  |
| **Family History of DM** | Present | 58 (79.5) | 15 (20.5) | 73 | 0.86 (0.38–1.94) | 0.719 | – | – |
|  | Absent | 50 (76.9) | 15 (23.1) | 65 | 1.00 |  |  |  |
| **Total** |  | 108 | 30 | 138 |  |  |  |  |

**STable 3: Association between Clinical Characteristics and Lifestyle Factors and Verbal Learning among Participants with Cognitive Impairment (n=138)**

| **Characteristics** | | **Verbal learning** | | **Total** | **COR (95% CI)** | **P value** | **AOR (95% CI)** | **P value** |
| --- | --- | --- | --- | --- | --- | --- | --- | --- |
|  |  | **Impaired** | **Intact** |  |  |  |  |  |
|  |  | **n(%)** | **n (%)** |  |  |  |  |  |
| **Recent RBS value mg/dL** | ≥200 | 59  (85.5) | 10  (14.5) | 69 | 2.41(1.03-5.63) | 0.042 | 2.35 (0.99–5.54) | 0.052 |
|  | <200 | 49  (71.0) | 20  (29.0) | 69 | 1.00 |  |  |  |
| **HbA1c(n=16)** | Good sugar control | 1  (50.0) | 1  (50.0) | 2 | 1.00 | 0.450 | - | - |
|  | Poor sugar control | 11  (78.6) | 3  (21.4) | 14 | 3.67 (0.173–77.6) |  |  |  |
| **Duration of DM (years)** | ≤10 | 82  (80.4) | 20  (19.6) | 102 | 1.00 | 0.307 |  |  |
|  | >10 | 26  (72.2) | 10  (27.8) | 36 | 0.634 (0.264–1.53) |  |  |  |
| **BMI(kg/m2)** | Normal/Underweight | 33  (68.8) | 15  (31.3) | 48 | 1.00 | 0.048 | 2.21 (0.96–5.10) | 0.064 |
|  | Overweight/Obese | 75  (83.3) | 15  (16.7) | 90 | 2.27 (0.996–5.18) |  |  |  |
| **Waist Hip Ratio** | Normal | 10  (71.4) | 4  (28.6) | 14 | 1.00 |  |  |  |
|  | High | 98  (79.0) | 26  (21.0) | 124 | 1.51 (0.44–5.20) | 0.516 |  |  |
| **Other Comorbidities among DM Patients** |  |  |  |  |  |  |  |  |
| **Hypertension** | Hypertensive | 51 (78.5) | 14 (21.5) | 65 | 1.02 (0.46–2.30) | 0.957 | – | – |
|  | Normotensive | 57 (78.1) | 16 (21.9) | 73 | 1.00 |  |  |  |
| **Hypercholesterolemia** | Present | 6 (85.7) | 1 (14.3) | 07 | 0.59 (0.07–5.07) | 0.997 | – | – |
|  | Absent | 102 (77.9) | 29 (22.1) | 131 | 1.00 |  |  |  |
| **Hypothyroidism** | Present | 3 (100.0) | 0 (0.0) | 03 | 2.02 (0.10–40.3) | 0.985 | – | – |
|  | Absent | 105 (77.8) | 30 (22.2) | 135 | 1.00 |  |  |  |
| **History of Physical Activity** | Yes | 27 (79.4) | 7 (20.6) | 34 | 1.00 |  | – | – |
|  | No | 81 (77.9) | 23 (22.1) | 104 | 0.91 (0.35–2.36) | 0.851 |  |  |
| **Alcohol Use** | Yes | 24 (80.0) | 6 (20.0) | 30 | 0.88 (0.32–2.39) | 0.794 | – | – |
|  | No | 84 (77.8) | 24 (22.2) | 108 | 1.00 |  |  |  |
| **Smoking Status** | Smoker | 19 (86.4) | 3 (13.6) | 22 | 0.52 (0.14–1.89) | 0.407 | – | – |
|  | Non-Smoker | 89 (76.7) | 27 (23.3) | 116 | 1.00 |  |  |  |
| **Tobacco Chewing** | Yes | 4 (80.0) | 1 (20.0) | 5 | 1.12 (0.12–10.4) | 0.997 | – | – |
|  | No | 104 (78.2) | 29 (21.8) | 133 | 1.00 |  |  |  |
| **Total** |  | 108 | 30 | 138 |  |  |  |  |

**STable 4: Association between sociodemographic variables and immediate verbal memory among participants with cognitive impairment (n=138)**

| **Characteristics** | | **Immediate Verbal Memory** | | **Total** | **COR (95% CI)** | **P value** | **AOR (95% CI)** | **P value** |
| --- | --- | --- | --- | --- | --- | --- | --- | --- |
|  |  | **Impaired** | **Intact** |  |  |  |  |  |
|  |  | **n (%)** | **n (%)** |  |  |  |  |  |
| **Age (years)** | <50 | 28  (100.0) | 0  (0.0) | 28 | 1.00 | 0.0  81 |  |  |
|  | ≥50 | 99  (90.0) | 11  (10.0) | 110 | 0.152(0.00868-2.66) |  |  |  |
| **Gender** | Male | 78  (92.9) | 6  (7.1) | 84 | 0.754 (0.218 – 2.60) | 0.7  51 |  |  |
|  | Female | 49  (90.7) | 5  (9.3) | 54 | 1.00 |  |  |  |
| **Education** | Up to class 10 | 81  (94.2) | 5  (5.8) | 86 | 2.11 (0.61–7.31) | 0.237 |  |  |
|  | Beyond Class 10 | 46  (88.5) | 6  (11.5) | 52 | 1.00 |  |  |  |
| **Occupation** | Professional | 30  (85.7) | 5  (14.3) | 35 | 1.00 | 0.141 | 1.00 |  |
|  | Skilled & Unskilled | 58  (96.7) | 2  (3.3) | 60 | 4.83(0.885-26.41) |  | 9.53 (1.28–71.12) | 0.028 |
|  | Homemaker/Unemployed | 39  (90.7) | 4  (9.3) | 43 | 1.63(0.401-6.58) |  | 5.48 (0.96–31.16) | 0.055 |
| **Socioeconomic class** | Upper | 41  (97.6) | 1  (2.4) | 42 | 1.00 | 0.198 | 5.93 | 0.118 |
|  | Middle | 41  (87.2) | 6  (12.8) | 47 | 0.167(0.0192 – 1.45) |  | 1.00 |  |
|  | Lower | 45  (91.8) | 4  (8.2) | 49 | 0.274(0.0295 – 2.56) |  | 1.94 | 0.370 |
| **Type of Family** | Nuclear | 99  (92.5) | 8  (7.5) | 107 | 1.00 | 0.7  10 |  |  |
|  | Joint | 28  (90.3) | 3  (9.7) | 31 | 0.754 (0.188 – 3.03) |  |  |  |
| **Marital Status** | Unmarried | 8 (100.0) | 0 (0.0) | 8 | - | - | – | – |
|  | Married | 119 (91.5) | 11 (8.5) | 130 | - |  |  |  |
| **Family History of DM** | Yes | 69 (94.5) | 4 (5.5) | 73 | 0.48 (0.13–1.72) | 0.252 | – | – |
|  | No | 58 (89.2) | 7 (10.8) | 65 | 1.00 | – | – | – |
| **Total** |  | 127 | 11 | 138 |  |  |  |  |

**STable 5: Association between clinical characteristics and lifestyle factors with immediate verbal memory among participants with cognitive impairment (n=138)**

| **Characteristics** | | **Immediate Verbal Memory** | | **Total** | **COR (95% CI)** | **P value** | **AOR (95% CI)** | **P value** |
| --- | --- | --- | --- | --- | --- | --- | --- | --- |
|  |  | **Impaired** | **Intact** |  |  |  |  |  |
|  |  | **n (%)** | **n (%)** |  |  |  |  |  |
| **Recent RBS value mg/dL** | ≥200 | 66  (95.7) | 3  (4.3) | 69 | 2.89(0.732-11.4) | 0.130 | 3.78 (0.81–17.69) | 0.091 |
|  | <200 | 61  (88.4) | 8  (11.6) | 69 | 1.00 |  | 1.00 |  |
| **HbA1c(n=16)** | Good sugar control | 2  (100.0) | 0  (0.0) | 2 | 1.00 | 0.997 |  |  |
|  | Poor sugar control | 13  (92.9) | 1  (7.1) | 14 | 1.80 (0.0558 – 58.0) |  |  |  |
| **Duration of DM (years)** | ≤10 | 94 (92.2) | 8 (7.8) | 102 | 1.00 | 0.989 |  |  |
|  | >10 | 33 (91.7) | 3 (8.3) | 36 | 0.94 (0.23–3.74) |  |  |  |
| **BMI (kg/m2)** | Normal/Underweight | 41  (85.4) | 7  (14.6) | 48 | 1.00 |  | 1.00 |  |
|  | Overweight/Obese | 86  (95.6) | 4  (4.4) | 90 | 3.67 (1.02 – 13.2) | 0.049 | 5.05 (1.10–23.08) | 0.037 |
| **Waist Hip Ratio** | Normal | 115  (92.7) | 9  (7.3) | 124 | 1.00 |  |  |  |
|  | High | 12  (85.7) | 2  (14.3) | 14 | 2.13 (0.41–11.0) | 0.3  67 |  |  |
| **Other Comorbidities among DM Patients** |  |  |  |  |  |  |  |  |
| **Hypertension** | Hypertensive | 59 (90.8) | 6 (9.2) | 65 | 0.72 (0.21–2.49) | 0.606 | – | – |
|  | Normotensive | 68 (93.2) | 5 (6.8) | 73 | 1.00 | – | – | – |
| **Hypercholesterolemia** | Present | 6 (85.7) | 1 (14.3) | 7 | 2.02 (0.22–18.4) | 0.448 | – | – |
|  | Absent | 121 (92.4) | 10 (7.6) | 131 | 1.00 | – | – | – |
| **Hypothyroidism** | Present | 3 (100.0) | 0 (0.0) | 3 | 0.65 (0.03–13.3) | 1.000 | – | – |
|  | Absent | 124 (91.9) | 11 (8.1) | 135 | 1.00 |  |  |  |
| **History of Physical Activity** | Yes | 32 (94.1) | 2 (5.9) | 34 | 1.00 |  |  |  |
|  | No | 95 (91.3) | 9 (8.7) | 104 | 0.66 (0.14–3.21) | 0.607 |  |  |
| **Alcohol Use** | Yes | 30 (100.0) | 0 (0.0) | 30 | 0.14 (0.008–2.43) | 0.121 | – | – |
|  | No | 97 (89.8) | 11 (10.2) | 108 | 1.00 | – | – | – |
| **Smoking Status** | Smoker | 21 (95.5) | 1 (4.5) | 22 | 0.51 (0.06–4.16) | 0.988 | – | – |
|  | Non-Smoker | 106 (91.4) | 10 (8.6) | 126 | 1.00 | – | – | – |
| **Tobacco Chewing** | Yes | 5 (100.0) | 0 (0.0) | 5 | 1.03 (0.05–19.9) | 1.000 | – | – |
|  | No | 122 (91.7) | 11 (8.3) | 133 | 1.00 | – | – | – |
| **Total** |  | 127 | 11 | 138 |  |  |  |  |

**STable 6: Association between sociodemographic variables and delayed verbal memory among participants with cognitive impairment (n=138)**

| **Characteristics** | | **Delayed Memory** | | **Total** | **COR (95% CI)** | **P value** | **AOR (95% CI)** | **P value** |
| --- | --- | --- | --- | --- | --- | --- | --- | --- |
|  |  | **Impaired** | **Intact** |  |  |  |  |  |
|  |  | **n (%)** | **n (%)** |  |  |  |  |  |
| **Age (years)** | <50 | 28  (100.0) | 0  (0.0) | 28 | 0.337 (0.0181 – 6.27) | 0.5  83 |  |  |
|  | ≥50 | 105  (95.5) | 5  (4.5) | 110 | 1.00 |  |  |  |
| **Gender** | Male | 84  (100.0) | 0  (0.0) | 84 | 1.00 | 0.0  08 |  |  |
|  | Female | 49  (90.7) | 5  (9.3) | 54 | 0.0533 (0.003 – 0.984) |  |  |  |
| **Education** | Up to class 10 | 82  (95.3) | 4  (4.7) | 86 | 0.40 (0.04–3.70) | 0.421 |  |  |
|  | Beyond Class 10 | 51  (98.1) | 1  (1.9) | 52 | 1.00 |  |  |  |
| **Occupation** | Professional | 9  (100.0) | 0  (0.0) | 9 |  | 1.00 |  |  |
|  | Skilled & Unskilled | 85  (98.8) | 1  (1.2) | 86 |  | 0.995 |  |  |
|  | Homemaker/Unemployed | 39  (90.7) | 4  (9.3) | 43 |  | 0.994 |  |  |
| **Socioeconomic class** | Upper | 40  (95.2) | 2  (4.8) | 42 | 1.00 |  |  |  |
|  | Middle | 45  (95.7) | 2  (4.3) | 47 | 1.12(0.151 – 8.36) | 0.908 |  |  |
|  | Lower | 40  (95.2) | 2  (4.8) | 42 | 2.40(0.210 – 27.45) | 0.481 |  |  |
| **Type of Family** | Nuclear | 102  (95.3) | 5  (4.7) | 107 |  | 0.5  87 |  |  |
|  | Joint | 31  (100.0) | 0  (0.0) | 31 |  |  |  |  |
| **Marital Status** | Unmarried | 8 (100.0) | 0 (0.0) | 8 | - | - | – |  |
|  | Married | 125 (96.2) | 5 (3.8) | 130 | - | - | – |  |
| **Family History of DM** | Yes | 71 (97.3) | 2 (2.7) | 73 | 0.58 (0.09–3.60) | 0.666 | – |  |
|  | No | 62 (95.4) | 3 (4.6) | 65 | 1.00 | – | – |  |
| **Total** |  | 133 | 5 | 138 |  |  |  |  |

**STable 7: Association between clinical characteristics and lifestyle factors with delayed verbal memory among participants with cognitive impairment (n=138)**

| **Characteristics** | | **Delayed Memory** | | **Total** | **COR (95% CI)** | **P value** | **AOR (95% CI)** | **P value** |
| --- | --- | --- | --- | --- | --- | --- | --- | --- |
|  |  | **Impaired** | **Intact** |  |  |  |  |  |
|  |  | **n (%)** | **n (%)** |  |  |  |  |  |
| **Recent RBS value mg/dL** | ≥200 | 68  (98.6) | 1  (1.4) | 69 | 4.18(0.456-38.4) | 0.206 | 3.44 (0.30–39.9) | 0.323 |
|  | <200 | 65  (94.2) | 4  (5.8) | 69 | 1.00 |  | 1.00 |  |
| **HbA1c(n=16)** | Good sugar control | 2  (100.0) | 0  (0.0) | 2 | 1.80 (0.0558 – 58.0) | 0.997 |  |  |
|  | Poor sugar control | 13  (92.9) | 1  (7.1) | 14 | 1.00 |  |  |  |
| **Duration of DM (years)** | ≤10 | 99  (97.1) | 3  (2.9) | 102 | 1.00 | 0.6  05 |  |  |
|  | >10 | 34  (94.4) | 2  (5.6) | 36 | 0.515 (0.0825 – 3.21) |  |  |  |
| **BMI(kg/m2)** | Normal/Underweight | 46  (95.8) | 2  (4.2) | 48 | 1.00 | 0.988 |  |  |
|  | Overweight/Obese | 87  (96.7) | 3  (3.3) | 90 | 1.26 (0.203 – 7.82) |  |  |  |
| **Waist Hip Ratio** | Normal | 13  (92.9) | 1  (7.1) | 14 | 1.00 |  |  |  |
|  | High | 120  (96.8) | 4  (3.2) | 124 | 0.433 (0.0450 – 4.17) | 0.4  19 |  |  |
| **Other Comorbidities among DM Patients** |  |  |  |  |  |  |  |  |
| **Hypertension** | Hypertensive | 60 (92.3) | 5 (7.7) | 65 | 0.07 (0.004–1.38) | 0.021 | – | – |
|  | Normotensive | 73 (100.0) | 0 (0.0) | 73 | 1.00 | – | – | – |
| **Hypercholesterolemia** | Present | 7 (100.0) | 0 (0.0) | 7 | 1.53 (0.08–30.4) | 0.997 | – | – |
|  | Absent | 126 (96.2) | 5 (3.8) | 131 | 1.00 | – | – | – |
| **Hypothyroidism** | Present | 3 (100.0) | 0 (0.0) | 3 | 0.30 (0.01–6.44) | 0.834 | – | – |
|  | Absent | 130 (96.3) | 5 (3.7) | 135 | 1.00 | – | – | – |
| **History of Physical Activity** | Yes | 34 (100.0) | 0 (0.0) | 34 | - | - |  |  |
|  | No | 99 (95.2) | 5 (4.8) | 104 | - | - |  |  |
| **Alcohol Use** | Yes | 30 (100.0) | 0 (0.0) | 30 | 0.31 (0.02–5.74) | 0.585 |  |  |
|  | No | 103 (95.4) | 5 (4.6) | 108 | 1.00 | – |  |  |
| **Smoking Status** | Smoker | 22 (100.0) | 0 (0.0) | 22 | 0.45 (0.02–8.44) | 0.997 | – | – |
|  | Non-Smoker | 111 (95.7) | 5 (4.3) | 116 | 1.00 | – | – | – |
| **Tobacco Chewing** | Yes | 5 (100.0) | 0 (0.0) | 5 | 0.47 (0.02–9.63) | 0.995 | – | – |
|  | No | 128 (96.2) | 5 (3.8) | 133 | 1.00 | – | – | – |
| **Total** |  | 133 | 5 | 138 |  |  |  |  |

**STable 8: Association between sociodemographic variables and recognition memory among participants with cognitive impairment (n=138)**

| **Characteristics** | | **Recognition** | | **Total** | **COR (95% CI)** | **P value** | **AOR (95% CI)** | **P value** |
| --- | --- | --- | --- | --- | --- | --- | --- | --- |
|  |  | **Impaired** | **Intact** |  |  |  |  |  |
|  |  | **n (%)** | **n (%)** |  |  |  |  |  |
| **Age (years)** | <50 | 26  (92.9) | 2  (7.1) | 28 | 1.00 | 0.735 | - | - |
|  | ≥50 | 97  (88.2) | 13  (11.8) | 110 | 0.574 (0.122–2.71) |  |  |  |
| **Gender** | Male | 77  (91.7) | 7  (8.3) | 84 | 0.523 (0.178–1.54) | 0.233 |  |  |
|  | Female | 46  (85.2) | 8  (14.8) | 54 | 1.00 |  |  |  |
| **Education** | Up to class 10 | 77  (89.5) | 9  (10.5) | 86 | 1.00 |  | - | - |
|  | Beyond Class 10 | 46  (88.5) | 6  (11.5) | 52 | 1.12 (0.37–3.34) | 0.844 |  |  |
| **Occupation** | Professional | 31  (88.6) | 4  (11.4) | 35 | 1.00 | 0.301 |  |  |
|  | Skilled & Unskilled | 56  (93.3) | 4  (6.7) | 60 | 2.72 (0.743 – 9.97) |  |  |  |
|  | Homemaker/Unemployed | 36  (83.7) | 7  (16.3) | 43 | 1.51 (0.403 – 5.64) |  |  |  |
| **Socioeconomic class** | Upper | 38  (90.5) | 4  (9.5) | 42 | 1.00 | 0.548 |  |  |
|  | Middle | 40  (85.1) | 7  (14.9) | 47 | 0.602 (0.163 – 2.22) |  |  |  |
|  | Lower | 45  (91.8) | 4  (8.2) | 49 | 1.184 (0.277 – 5.06) |  |  |  |
| **Type of Family** | Nuclear | 98  (91.6) | 9  (8.4) | 107 | 0.383 (0.125–1.18) | 0.085 | 2.70 (0.87–8.42) | 0.086 |
|  | Joint | 25  (80.6) | 6  (19.4) | 31 | 1.00 |  | 1.00 |  |
| **Marital Status** | Unmarried | 8 (100.0) | 0 (0.0) | 8 | - |  | – | – |
|  | Married | 115 (88.5) | 15 (11.5) | 130 | - |  | – | – |
| **Family History of DM** | Yes | 67 (91.8) | 6 (8.2) | 73 | 0.56 (0.19–1.66) | 0.289 | – |  |
|  | No | 56 (86.2) | 9 (13.8) | 65 | 1.00 | – | – |  |
| **Total** |  | 123 | 15 | 138 |  |  |  |  |

**STable 9: Association between Clinical Characteristics and Lifestyle Factors and Recognition Memory among Participants with Cognitive Impairment (n=138)**

| **Characteristics** | | | | **Recognition** | | | | **Total** | **COR (95% CI)** | **P value** | **AOR (95% CI)** | **P value** | |
| --- | --- | --- | --- | --- | --- | --- | --- | --- | --- | --- | --- | --- | --- |
|  |  |  |  | **Impaired** | | **Intact** | |  |  |  |  |  |  |
|  |  |  |  | **n (%)** | | **n (%)** | |  |  |  |  |  |  |
| **Recent RBS value mg/dL** | | ≥200 | | 64  (92.8) | | 5  (7.2) | | 69 | 0.461 (0.149–1.43) | 0.171 | 2.25 (0.72–7.06) | 0.166 | |
|  |  | <200 | | 59  (85.5) | | 10  (14.5) | | 69 | 1.00 |  | 1.00 |  | |
| **HbA1c(n=16)** | | Good sugar control | | 1  (50.0) | | 1  (50.0) | | 2 | 1.00 | 0.242 | - | - | |
|  | | Poor sugar control | | 13  (92.9) | | 1  (7.1) | | 14 | 13.0 (0.418–405) |  |  |  | |
| **Duration of DM (years)** | | ≤10 | | 93  (91.2) | | 9  (8.8) | | 102 | 1.00 | 0.218 |  |  | |
|  |  | >10 | | 30  (83.3) | | 6  (16.7) | | 36 | 0.484 (0.159–1.47) |  |  |  | |
| **BMI(kg/m2)** | | Normal/Underweight | | 43  (89.6) | | 5  (10.4) | | 48 | 1.00 | 0.901 |  |  | |
|  |  | Overweight/Obese | | 80  (88.9) | | 10  (11.1) | | 90 | 0.930 (0.299–2.90) |  |  |  | |
| **Waist Hip Ratio** | | Normal | | 12  (85.7) | | 2  (14.3) | | 14 | 1.00 |  |  |  | |
|  |  | High | | 111  (89.5) | | 13  (10.5) | | 124 | 1.42 (0.29–7.07) | 0.666 |  |  | |
| **Other Comorbidities among DM Patients** | |  | |  | |  | |  |  |  |  |  | |
| **Hypertension** | | Hypertensive | | 56 (86.2) | | 9 (13.8) | | 65 | 0.56 (0.19–1.66) | 0.289 | – | – | |
|  |  | Normotensive | | 67 (91.8) | | 6 (8.2) | | 73 | 1.00 | – | – | – | |
| **Hypercholesterolemia** | | Present | | 6 (85.7) | | 1 (14.3) | | 7 | 1.39 (0.16–12.4) | 0.562 | – | – | |
|  |  | Absent | | 117 (89.3) | | 14 (10.7) | | 131 | 1.00 | – | – | – | |
| **Hypothyroidism** | | Present | | 3 (100.0) | | 0 (0.0) | | 3 | 0.90 (0.04–18.3) | 0.997 | – | – | |
|  |  | Absent | | 120 (88.9) | | 15 (11.1) | | 135 | 1.00 | – | – | – | |
| **History of Physical Activity** | | Yes | | 31 (91.2) | | 3 (8.8) | | 34 | 1.00 |  | – | – | |
|  | | No | | 92 (88.5) | | 12 (11.5) | | 104 | 0.74 (0.20–2.80) | 0.660 | – | – | |
| **Alcohol Use** | Yes | | 29 (96.7) | | 1 (3.3) | | 30 | | 0.23 (0.03–1.84) | 0.191 | – | – |  |
|  | No | | 94 (87.0) | | 14 (13.0) | | 108 | | 1.00 | – | – | – |  |
| **Smoking Status** | Smoker | | 22 (100.0) | | 0 (0.0) | | 22 | | 0.15 (0.01–2.52) | 0.128 | – | – |  |
|  | Non-Smoker | | 101 (87.1) | | 15 (12.9) | | 116 | | 1.00 | – | – | – |  |
| **Tobacco Chewing** | Yes | | 4 (80.0) | | 1 (20.0) | | 5 | | 0.47 (0.05–4.51) | 0.443 | – | – |  |
|  | No | | 119 (89.5) | | 14 (10.5) | | 133 | | 1.00 | – | – | – |  |
| **Total** |  | | 123 | | 15 | | 138 | |  |  |  |  |  |

**STable 10: Association between sociodemographic variables and planning- and problem-solving impairment among participants with cognitive impairment (n = 138)**

| **Characteristics** | | **Planning and problem solving** | | **Total** | **COR (95% CI)** | **P value** | **AOR (95% CI)** | **P value** |
| --- | --- | --- | --- | --- | --- | --- | --- | --- |
|  |  | **Impaired** | **Intact** |  |  |  |  |  |
|  |  | **n (%)** | **n (%)** |  |  |  |  |  |
| **Age (years)** | <50 | 3  (10.7) | 25  (89.3) | 28 | 1.02 (0.267–3.89) | 0.976 | - |  |
|  | ≥50 | 12  (10.9) | 98  (89.1) | 110 | 1.00 |  |  |  |
| **Gender** | Male | 8  (9.5) | 76  (90.5) | 84 | 1.41 (0.482–4.16) | 0.526 | - |  |
|  | Female | 7  (13.0) | 47  (87.0) | 54 | 1.00 |  |  |  |
| **Education** | Up to class 10 | 11  (12.8) | 75  (87.2) | 86 | 1.76 (0.53–5.85) | 0.356 | - |  |
|  | Beyond Class 10 | 4  (7.7) | 48  (92.3) | 52 | 1.00 |  |  |  |
| **Occupation** | Professional | 2  (5.7) | 33  (94.3) | 35 | 1.00 | 0.537 |  |  |
|  | Skilled & Unskilled | 8  (13.3) | 52  (86.7) | 60 | 2.539 (0.507– 12.69) |  |  |  |
|  | Homemaker/Unemployed | 5  (11.6) | 38  (88.4) | 43 | 2.171 (0.394 – 11.942) |  |  |  |
| **Socioeconomic class** | Upper | 4  (9.5) | 38  (90.5) | 42 | 1.00 | 0.661 |  |  |
|  | Middle | 4  (8.5) | 43  (91.5) | 47 | 0.88 (0.206– 3.779) |  |  |  |
|  | Lower | 7  (14.3) | 42  (85.7) | 49 | 1.58 (0.429 – 5.836) |  |  |  |
| **Type of Family** | Nuclear | 9  (8.4) | 98  (91.6) | 107 | 1.00 | 0.103 | 0.31 (0.09–1.02) | 0.053 |
|  | Joint | 6  (19.4) | 25  (80.6) | 31 | 2.61 (0.851–8.03) |  | 1.00 |  |
| **Marital Status** | Married | 15(11.5) | 115(88.5) | 130 | 1.00 |  |  |  |
|  | Unmarried | 0(0.0) | 8(100.0) | 8 | 0.438(0.0241-7.797) | 0.598 |  |  |
| **Family History of DM** | Absent | 11 (16.9%) | 54 (83.1%) | 65 | 3.51 (1.06–11.6) | 0.031 | 4.07(1.87-14.015) | 0.026 |
|  | Present | 4 (5.5%) | 69 (94.5%) | 73 | 1.00 |  | 1.00 |  |
| **Total** |  | 15 | 123 | 138 |  |  |  |  |

**STable 11: Association between Clinical Characteristics and Lifestyle Factors with Planning & Problem-Solving Impairment among Participants with Cognitive Impairment (n = 138)**

| **Characteristics** | | **Planning and problem solving** | | **Total** | **COR (95% CI)** | **P value** | **AOR (95% CI)** | **P value** |
| --- | --- | --- | --- | --- | --- | --- | --- | --- |
|  |  | **Impaired** | **Intact** |  |  |  |  |  |
|  |  | **n (%)** | **n (%)** |  |  |  |  |  |
| **Recent RBS value mg/dL** | ≥200 | 7  (10.1) | 62  (89.9) | 69 | 1.16 (0.397–3.40) | 0.784 |  |  |
|  | <200 | 8  (11.6) | 61  (88.4) | 69 | 1.00 |  |  |  |
| **HbA1c(n=16)** | Good sugar control | 0  (0.0) | 2  (100.0) | 2 | 1.00 | 0.997 | - | - |
|  | Poor sugar control | 1  (7.1) | 13  (92.9) | 14 | 0.556 (0.0172–17.9) |  |  |  |
| **Duration of DM (years)** | ≤10 | 13  (12.7) | 89  (87.3) | 102 | 1.00 | 0.353 | - | - |
|  | >10 | 2  (5.6) | 34  (94.4) | 36 | 0.403 (0.0863–1.88) |  |  |  |
| **BMI(kg/m2)** | Normal/Underweight | 4  (8.3) | 44  (91.7) | 48 | 1.00 | 0.576 | - | - |
|  | Overweight/Obese | 11  (12.2) | 79  (87.8) | 90 | 1.53 (0.460–5.10) |  |  |  |
| **Waist Hip Ratio** | Normal | 1  (7.1) | 13  (92.9) | 14 | 1.00 |  | - | - |
|  | High | 14  (11.3) | 110  (88.7) | 14 | 1.65 (0.20–13.63) | 0.640 |  |  |
| **Other Comorbidities among DM Patients** |  |  |  |  |  |  |  |  |
| **Hypertension** | Hypertensive | 8 (12.3) | 57 (87.7) | 65 | 1.32 (0.45–3.88) | 0.785 |  |  |
|  | Normotensive | 7 (9.6) | 66 (90.4) | 73 | 1.00 |  |  |  |
| **Hypercholesterolemia** | Present | 0 (0.0) | 7 (100.0) | 7 | 2.00 (0.11–36.7) | 0.968 |  |  |
|  | Absent | 15 (11.5) | 116 (88.5) | 131 | 1.00 |  |  |  |
| **Hypothyroidism** | Present | 0 (0.0) | 3 (100.0) | 3 | 1.11 (0.05–22.5) | 0.997 |  |  |
|  | Absent | 15 (11.1) | 120 (88.9) | 135 | 1.00 |  |  |  |
| **History of Physical Activity** | Yes | 4 (11.8) | 30 (88.2) | 34 | 1.00 |  |  |  |
|  | No | 11 (10.6) | 93 (89.4) | 104 | 0.89 (0.26–2.99), | 0.847 |  |  |
| **Alcohol Use** | Yes | 1 (3.3) | 29 (96.7) | 30 | 4.32 (0.54–34.3) | 0.191 | 0.23 (0.03–1.91) | 0.174 |
|  | No | 14 (13.0) | 94 (87.0) | 108 | 1.00 |  | 1.00 |  |
| **Smoking Status** | Smoker | 1 (4.5) | 21 (95.5) | 22 | 2.88 (0.36–23.1) | 0.465 |  |  |
|  | Non-Smoker | 14 (12.1) | 102 (87.9) | 116 | 1.00 |  |  |  |
| **Tobacco Chewing** | Yes | 1 (20.0) | 4 (80.0) | 5 | 2.13 (0.22–20.4) | 0.443 |  |  |
|  | No | 14 (10.5) | 119 (89.5) | 133 | 1.00 |  |  |  |
| **Total** |  | 15 | 123 | 138 |  |  |  |  |

**STable 12: Association between sociodemographic variables and sustained attention (processing speed) impairment among participants with cognitive impairment (n = 138)**

| **Characteristics** | | **Sustained Attention (Processing Speed)** | | **Total** | **COR (95% CI)** | **P value** | **AOR (95% CI)** | **P value** |
| --- | --- | --- | --- | --- | --- | --- | --- | --- |
|  |  | **Impaired** | **Intact** |  |  |  |  |  |
|  |  | **n (%)** | **n (%)** |  |  |  |  |  |
| **Age (years)** | <50 | 21  (75.0) | 7  (25.0) | 28 | 0.778 (0.302–2.01) | 0.603 |  |  |
|  | ≥50 | 77  (70.0) | 33  (30.0) | 110 | 1.00 |  |  |  |
| **Gender** | Male | 56  (66.7) | 28  (33.3) | 84 | 1.75 (0.798–3.84) | 0.160 | 1.00 |  |
|  | Female | 42  (77.8) | 12  (22.2) | 54 | 1.00 |  | 1.71 (0.76–3.83) | 0.194 |
| **Education** | Up to class 10 | 61  (70.9) | 25  (29.1) | 86 | 1.00 |  |  |  |
|  | Beyond Class 10 | 37  (71.2) | 15  (28.8) | 52 | 0.99 (0.46–2.11) | 0.978 |  |  |
| **Occupation** | Professional | 23  (65.7) | 12  (34.3) | 35 | 1.00 | 0.719 |  |  |
|  | Skilled & Unskilled | 44  (73.3) | 16  (26.7) | 60 | 1.43 (0.582 – 3.54) |  |  |  |
|  | Homemaker/Unemployed | 31  (72.1) | 12  (27.9) | 43 | 1.35 (0.514 – 3.54) |  | - |  |
| **Socioeconomic class** | Upper | 24  (57.1) | 18  (42.9) | 42 | 1.00 |  | 1.00 |  |
|  | Middle | 39  (83.0) | 8  (17.0) | 47 | 3.66 (1.378 – 9.70) | 0.009 | 3.59 (1.35–9.59) | 0.011 |
|  | Lower | 35  (71.4) | 14  (28.6) | 49 | 1.87 (0.785 – 4.48) | 0.157 | 1.93 (0.80–4.64) | 0.143 |
| **Type of Family** | Nuclear | 74  (69.2) | 33  (30.8) | 107 | 1.53 (0.599–3.90) | 0.372 |  |  |
|  | Joint | 24  (77.4) | 7  (22.6) | 31 | 1.00 |  |  |  |
| **Marital Status** | Unmarried | 5 (62.5) | 3 (37.5) | 8 | 0.66 (0.15–2.92) | 0.691 |  |  |
|  | Married | 93 (71.5) | 37 (28.5) | 130 | 1.00 |  |  |  |
| **Family History of DM** | Absent | 48 (73.8) | 17 (26.2) | 65 | 1.00 |  |  |  |
|  | Present | 50 (68.5) | 23 (31.5) | 73 | 1.30 (0.62–2.73) | 0.574 |  |  |
| **Total** |  | 98 | 40 | 138 |  |  |  |  |

**STable 13: Association between Clinical Characteristics and Lifestyle Factors and Sustained Attention (Processing Speed) Impairment among Participants with Cognitive Impairment (n = 138)**

| **Characteristics** | | **Sustained Attention (Processing Speed)** | | **Total** | | **COR (95% CI)** | **P-value** | **AOR (95% CI)** | **P value** |
| --- | --- | --- | --- | --- | --- | --- | --- | --- | --- |
|  |  | **Impaired** | **Intact** |  |  |  |  |  |  |
|  |  | **n (%)** | **n (%)** |  | |  |  |  |  |
| **Recent RBS value (mg/dL)** | ≥200 | 44 (63.8) | 25 (36.2) | 69 | | 2.05(0.963-4.35) | 0.061 |  |  |
|  | <200 | 15 (21.7) | 54 (78.3) | 69 | |  |  |  |  |
| **HbA1c(n=16)** | Good sugar control | 2  (100.0) | 0  (0.0) | 2 | | 1.00 | 0.997 |  |  |
|  | Poor sugar control | 9  (64.3) | 5  (35.7) | 14 | | 0.345 (0.0139–8.58) |  |  |  |
| **Duration of DM (years)** | ≤10 | 74  (72.5) | 28  (27.5) | 102 | | 0.757 (0.334–1.71) | 0.504 |  |  |
|  | >10 | 24  (66.7) | 12  (33.3) | 36 | | 1.00 |  |  |  |
| **BMI(kg/m2)** | Normal/Underweight | 35  (72.9) | 13  (27.1) | 48 | | 0.867 (0.397–1.89) | 0.719 |  |  |
|  | Overweight/Obese | 63  (70.0) | 27  (30.0) | 90 | | 1.00 |  |  |  |
| **Waist Hip Ratio** | Normal | 9  (64.3) | 5  (35.7) | 14 | | 1.00 |  |  |  |
|  | High | 89  (71.8) | 35  (28.2) | 124 | | 1.41 (0.44–4.51 | 0.560 |  |  |
| **Other Co-morbidities among DM Patients** |  |  |  |  | |  |  |  |  |
| **Hypertension** | Hypertensive | 49 (75.4) | 16 (24.6) | 65 | 1.50 (0.71–3.16) | | 0.286 |  |  |
|  | Normotensive | 49 (67.1) | 24 (32.9) | 73 | 1.00 | |  |  |  |
| **Hypercholesterolemia** | Present | 7 (100.0) | 0 (0.0) | 7 | 0.15 (0.008–2.70) | | 0.107 |  |  |
|  | Absent | 91 (69.5) | 40 (30.5) | 131 | 1.00 | |  |  |  |
| **Hypothyroidism** | Present | 2 (66.7) | 1 (33.3) | 3 | 0.81 (0.072–9.22) | | 0.985 |  |  |
|  | Absent | 96 (71.1) | 39 (28.9) | 135 | 1.00 | |  |  |  |
| **History of Physical Activity** | Yes | 22(64.7) | 12(35.3) | 34 | 1.00 | |  |  |  |
|  | No | 76(73.1) | 28(26.9) | 104 | 1.48 (0.65–3.38) | | 0.357 |  |  |
| **Alcohol Use** | Yes | 20 (66.7) | 10 (33.3) | 30 | 1.30 (0.55–3.10) | | 0.553 |  |  |
|  | No | 78 (72.2) | 30 (27.8) | 108 | 1.00 | |  |  |  |
| **Smoking Status** | Smoker | 18 (81.8) | 4 (18.2) | 22 | 0.49 (0.16–1.56) | | 0.307 |  |  |
|  | Non-Smoker | 80 (69.0) | 36 (31.0) | 116 | 1.00 | |  |  |  |
| **Tobacco Chewing** | Yes | 4 (80.0) | 1 (20.0) | 5 | 1.66 (0.18–15.3) | | 0.997 |  |  |
|  | No | 94 (70.7) | 39 (29.3) | 133 | 1.00 | |  |  |  |
| **Total** |  | 98 | 40 | 138 |  | |  |  |  |

**STable 14: Association between sociodemographic variables and sustained attention (accuracy) impairment among participants with cognitive impairment (n = 138)**

| **Characteristics** | | **Sustained Attention (Accuracy)** | | **Total** | **COR (95% CI)** | | **P value** | **AOR (95% CI)** | **P value** |
| --- | --- | --- | --- | --- | --- | --- | --- | --- | --- |
|  |  | **Impaired** | **Intact** |  |  |  |  |  |  |
|  |  | **n (%)** | **n (%)** |  |  |  |  |  |  |
| **Age (years)** | <50 | 20  (71.4) | 8  (28.6) | 28 | 1.00 | | 0.058 | 1.00 |  |
|  | ≥50 | 95  (86.4) | 15  (13.6) | 110 | 2.53 (0.947–6.78) | |  | 2.69 (0.96 – 7.56) | 0.061 |
| **Gender** | Male | 69  (82.1) | 15  (17.9) | 84 | 1.25 (0.490–3.19) | | 0.640 |  |  |
|  | Female | 46  (85.2) | 8  (14.8) | 54 | 1.00 | |  |  |  |
| **Education** | Up to class 10 | 71  (82.6) | 15  (17.4) | 86 | 1.16 (0.455–2.97) | | 0.753 |  |  |
|  | Beyond Class 10 | 44  (84.6) | 8  (15.4) | 52 | 1.00 | |  |  |  |
| **Occupation** | Professional | 30  (85.7) | 5  (14.3) | 35 | 1.00 | | 0.366 |  |  |
|  | Skilled & Unskilled | 47  (78.3) | 13  (21.7) | 60 | 0.60 (0.195 – 1.86) | |  |  |  |
|  | Homemaker/Unemployed | 38  (88.4) | 5  (11.6) | 43 | 1.27 (0.335 – 4.78) | |  |  |  |
| **Socioeconomic class** | Upper | 34  (81.0) | 8  (19.0) | 42 | 1.00 | | 0.388 |  |  |
|  | Middle | 42  (89.4) | 5  (10.6) | 47 | 1.98 (0.592 – 6.60) |  | |  |  |
|  | Lower | 39  (79.6) | 10  (20.4) | 49 | 0.92 (0.325 – 2.59) |  | |  |  |
| **Type of Family** | Nuclear | 89  (83.2) | 18  (16.8) | 107 | 1.05 (0.356–3.11) | 0.927 | | 1.38 (0.44 – 4.35) | 0.578 |
|  | Joint | 26  (83.9) | 5  (16.1) | 31 | 1.00 |  | | 1.00 |  |
| **Marital Status** | Unmarried | 4 (50.0%) | 4 (50.0%) | 8 | 1.00 |  | | 1.00 |  |
|  | Married | 111 (85.4%) | 19 (14.6%) | 130 | 0.171 (0.039–0.744) | 0.009 | | 8.4(1.6-44.5) | 0.012 |
| **Family History of DM** | Absent | 60 (92.3) | 5 (7.7) | 65 | 3.93 (1.37–11.3) | 0.008 | | 4.38(1.43-13.43) | 0.010 |
|  | Present | 55 (75.3) | 18 (24.7) | 73 | 1.00 |  | | 1.00 |  |
| **Total** |  | 115 | 23 | 138 |  |  | |  |  |

**STable 15: Association between Clinical Characteristics and Lifestyle Factors and Sustained Attention (Accuracy) Impairment among Participants with Cognitive Impairment (n = 138)**

| **Characteristics** | | **Sustained Attention (Accuracy)** | | **Total** | **COR (95% CI)** | **P value** | **AOR (95% CI)** | **P value** |
| --- | --- | --- | --- | --- | --- | --- | --- | --- |
|  |  | **Impaired** | **Intact** |  |  |  |  |  |
|  |  | **n (%)** | **n (%)** |  |  |  |  |  |
| **Recent RBS value mg/dL** | ≥200 | 55  (79.7) | 14  (20.3) | 69 | 1.70 (0.680–4.23) | 0.253 |  |  |
|  | <200 | 60  (87.0) | 9  (13.0) | 69 | 1.00 |  |  |  |
| **HbA1c(n=16)** | Good sugar control | 2  (100.0) | 0  (0.0) | 2 | 0.657 (0.0251–17.2) | 0.997 |  |  |
|  | Poor sugar control | 11  (78.6) | 3  (21.4) | 14 | - |  |  |  |
| **Duration of DM (years)** | ≤10 | 86  (84.3) | 16  (15.7) | 102 | 0.771 (0.288–2.06) | 0.603 |  |  |
|  | >10 | 29  (80.6) | 7  (19.4) | 36 | 1.00 |  |  |  |
| **BMI(kg/m2)** | Normal/Underweight | 41  (85.4) | 7  (14.6) | 48 | 0.790 (0.300–2.08) | 0.632 |  |  |
|  | Overweight/Obese | 74  (82.2) | 16  (17.8) | 90 | 1.00 |  |  |  |
| **Waist Hip Ratio** | Normal | 13  (92.9) | 1  (7.1) | 14 | 1.00 |  |  |  |
|  | High | 102  (82.3) | 22  (17.7) | 124 | 2.80 (0.348–22.6) | 0.465 |  |  |
| **History of Physical Activity** | Yes | 26 (76.5) | 8 (23.5) | 34 | 1.00 |  |  |  |
|  | No | 89 (85.6) | 15 (14.4) | 104 | 1.83 (0.70–4.78) | 0.220 |  |  |
| **Other Comorbidities among DM Patients** |  |  |  |  |  |  |  |  |
| **Hypertension** | Hypertensive | 56 (86.2) | 9 (13.8) | 65 | 1.48 (0.592–3.68) | 0.402 |  |  |
|  | Normotensive | 59 (80.8) | 14 (19.2) | 73 | 1.00 |  |  |  |
| **Hypercholesterolemia** | Present | 6 (85.7) | 1 (14.3) | 7 | 0.83 (0.09–7.20) | 0.997 |  |  |
|  | Absent | 109 (83.2) | 22 (16.8) | 131 | 1.00 |  |  |  |
| **Hypothyroidism** | Present | 3 (100.0) | 0 (0.0) | 3 | 1.46 (0.073–29.3) | 0.985 |  |  |
|  | Absent | 112 (83.0) | 23 (17.0) | 135 | 1.00 |  |  |  |
| **Alcohol Use** | Yes | 24 (80.0) | 6 (20.0) | 30 | 1.34 (0.476–3.76) | 0.580 |  |  |
|  | No | 91 (84.3) | 17 (15.7) | 108 | 1.00 |  |  |  |
| **Smoking Status** | Smoker | 17 (77.3) | 5 (22.7) | 22 | 1.60 (0.524–4.89) | 0.405 |  |  |
|  | Non-Smoker | 98 (84.5) | 18 (15.5) | 116 | 1.00 |  |  |  |
| **Tobacco Chewing** | Yes | 4 (80.0) | 1 (20.0) | 5 | 0.793 (0.0845–7.44) | 0.997 |  |  |
|  | No | 111 (83.5) | 22 (16.5) | 133 | 1.00 |  |  |  |
| **Total** |  | 115 | 23 | 138 |  |  |  |  |

**STable 16: Association between sociodemographic variables and focused attention impairment among participants with cognitive impairment (n = 138)**

| **Characteristics** | | **Focused Attention** | | **Total** | **COR (95% CI)** | **P value** | **AOR (95% CI)** | **P value** |
| --- | --- | --- | --- | --- | --- | --- | --- | --- |
|  |  | **Impaired** | **Intact** |  |  |  |  |  |
|  |  | **n (%)** | **n(%)** |  |  |  |  |  |
| **Age (years)** | <50 | 3  (10.7) | 25  (89.3) | 28 | 1.22 (0.324–4.56) | 0.995 |  |  |
|  | ≥50 | 14  (12.7) | 96  (87.3) | 110 | 1.00 |  |  |  |
| **Gender** | Male | 9  (10.7) | 75  (89.3) | 84 | 1.45 (0.522–4.02) | 0.474 |  |  |
|  | Female | 8  (14.8) | 46  (85.2) | 54 | 1.00 |  |  |  |
| **Education** | Up to class 10 | 12  (14.0) | 74  (86.0) | 86 | 0.656 (0.217–1.98) | 0.452 |  |  |
|  | Beyond Class 10 | 5  (9.6) | 47  (90.4) | 52 | 1.00 |  |  |  |
| **Occupation** | Professional | 2  (5.7) | 33  (94.3) | 35 | 1.00 | 0.134 | 1.00 |  |
|  | Skilled & Unskilled | 6  (10.0) | 54  (90.0) | 60 | 1.83 (0.349 – 9.62) |  | 1.27 (0.22–7.34) | 0.786 |
|  | Homemaker/Unemployed | 9  (20.9) | 34  (79.1) | 43 | 4.37 (0.877 – 21.74) |  | 4.03 (0.74–21.96) | 0.107 |
| **Socioeconomic class** | Upper | 1  (2.4) | 41  (97.6) | 42 | 1.00 | 0.046 | 1.00 |  |
|  | Middle | 8  (17.0) | 39  (83.0) | 47 | 8.00 (0.95 – 66.87) |  | 8.24 (0.93–73.09) | 0.058 |
|  | Lower | 8  (16.3) | 41  (83.7) | 49 | 8.41 (1.00 – 70.38) |  | 8.96 (1.01–81.85) | 0.050 |
| **Type of Family** | Nuclear | 12  (11.2) | 95  (88.8) | 107 | 1.52 (0.492–4.71) | 0.535 |  |  |
|  | Joint | 5  (16.1) | 26  (83.9) | 31 | 1.00 |  |  |  |
| **Marital Status** | Unmarried | 1 (12.5) | 7 (87.5) | 8 | 1.02 (0.117–8.82) | 0.997 |  |  |
|  | Married | 16 (12.3) | 114 (87.7) | 130 | 1.00 |  |  |  |
| **Family History of DM** | Absent | 8 (12.3) | 57 (87.7) | 65 | 1.00 |  |  |  |
|  | Present | 9 (12.3) | 64 (87.7) | 73 | 0.998 (0.361–2.76) | 0.997 |  |  |
| **Total** |  | 17 | 121 | 138 |  |  |  |  |

**STable 17: Association between Clinical Characteristics and Lifestyle Factors and Focused Attention Impairment among Participants with Cognitive Impairment (n = 138)**

| **Characteristics** | | **Focused Attention** | | **Total** | **COR (95% CI)** | **P value** | **AOR (95% CI)** | **P value** |
| --- | --- | --- | --- | --- | --- | --- | --- | --- |
|  |  | **Impaired** | **Intact** |  |  |  |  |  |
|  |  | **n (%)** | **n (%)** |  |  |  |  |  |
| **Recent RBS value mg/dL** | ≥200 | 8  (11.6) | 61  (88.4) | 69 | 1.14 (0.414–3.16) | 0.796 |  |  |
|  | <200 | 9  (13.0) | 60  (87.0) | 69 | 1.00 |  |  |  |
| **HbA1c(n=16)** | Good sugar control | 1  (50.0) | 1  (50.0) | 2 | 0.167 (0.00714–3.89) | 0.350 |  |  |
|  | Poor sugar control | 2  (14.3) | 12  (85.7) | 14 | 1.00 |  |  |  |
| **Duration of DM (years)** | ≤10 | 10  (9.8) | 92  (90.2) | 102 | 1.00 |  | 1.00 |  |
|  | >10 | 7  (19.4) | 29  (80.6) | 36 | 2.22 (0.775–6.36) | 0.146 | 2.93 (0.93–9.21) | 0.066 |
| **BMI(kg/m2)** | Normal/Underweight | 7  (14.6) | 41  (85.4) | 48 | 0.732 (0.260–2.06) | 0.554 |  |  |
|  | Overweight/Obese | 10  (11.1) | 80  (88.9) | 90 | 1.00 |  |  |  |
| **Waist Hip Ratio** | Normal | 16  (12.9) | 108  (87.1) | 124 | 1.00 | 0.997 |  |  |
|  | High | 1  (7.1) | 13  (92.9) | 14 | 0.519 (0.0635–4.24) |  |  |  |
| **Other Comorbidities among DM Patients** |  |  |  |  |  |  |  |  |
| **Hypertension** | Hypertensive | 8 (12.3) | 57 (87.7) | 65 | 0.998 (0.361–2.76) | 0.997 |  |  |
|  | Normotensive | 9 (12.3) | 64 (87.7) | 73 | 1.00 |  |  |  |
| **Hypercholesterolemia** | Present | 1 (14.3) | 6 (85.7) | 7 | 0.835 (0.094–7.39) | 0.985 |  |  |
|  | Absent | 16(12.2) | 115(87.8) | 131 | 1.00 |  |  |  |
| **Hypothyroidism** | Present | 0 (0.0) | 3 (100.0) | 3 | 0.967 (0.048–19.5) | 0.997 |  |  |
|  | Absent | 17 (12.6) | 118 (87.4) | 135 | 1.00 |  |  |  |

| **History of Physical Activity** | Yes | 2 (5.9) | 32 (94.1) | 34 | 1.00 |  |  |  |
| --- | --- | --- | --- | --- | --- | --- | --- | --- |
|  | No | 15 (14.4) | 89 (85.6) | 104 | 2.70 (0.58–12.45) | 0.241 |  |  |
| **Alcohol Use** | Yes | 4 (13.3) | 26 (86.7) | 30 | 0.889 (0.267–2.96) | 0.764 |  |  |
|  | No | 13 (12.0) | 95 (88.0) | 108 | 1.00 |  |  |  |
| **Smoking Status** | Smoker | 4 (18.2) | 18 (81.8) | 22 | 0.568 (0.166–1.94) | 0.476 |  |  |
|  | Non-Smoker | 13 (11.2) | 103 (88.8) | 116 | 1.00 |  |  |  |
| **Tobacco Chewing** | Yes | 0 (0.0) | 5 (100.0) | 5 | 0.605 (0.032–11.4) | 0.995 |  |  |
|  | No | 17 (12.8) | 116 (87.2) | 133 | 1.00 |  |  |  |
| **Total** |  | 17 | 121 | 138 |  |  |  |  |
